# Active Inference, homeostatic regulation and adaptive behavioural control

**DOI:** 10.1016/j.pneurobio.2015.09.001

**Published:** 2015-11

**Authors:** Giovanni Pezzulo, Francesco Rigoli, Karl Friston

**Affiliations:** a Institute of Cognitive Sciences and Technologies, National Research Council, Rome, Italy; b Wellcome Trust, UCL, London, UK

**Keywords:** CS, conditioned stimuli, US, unconditioned stimulus, PFC, prefrontal cortex, SMA, supplemental motor area, IC, inferotemporal cortex, AIC, anterior insular cortex, PMC/MC, premotor/motor cortex, ipoT, ipothalamus, ANS, autonomous nervous system, ACC, anterior cingulate cortex, PPC, posterior parietal cortex, VTA/SN, the dopaminergic ventral tegmental area and substantia nigra, Active Inference, Homeostatic regulation, Adaptive control, Model-based control, Model-free control, Pavlovian control

## Abstract

We review a theory of homeostatic regulation and adaptive behavioural control within the Active Inference framework. Our aim is to connect two research streams that are usually considered independently; namely, Active Inference and associative learning theories of animal behaviour. The former uses a probabilistic (Bayesian) formulation of perception and action, while the latter calls on multiple (Pavlovian, habitual, goal-directed) processes for homeostatic and behavioural control. We offer a synthesis these classical processes and cast them as successive hierarchical contextualisations of sensorimotor constructs, using the generative models that underpin Active Inference. This dissolves any apparent mechanistic distinction between the optimization processes that mediate classical control or learning. Furthermore, we generalize the scope of Active Inference by emphasizing interoceptive inference and homeostatic regulation. The ensuing homeostatic (or allostatic) perspective provides an intuitive explanation for how priors act as drives or goals to enslave action, and emphasises the embodied nature of inference.

## Highlights

• An Active Inference account of homeostatic regulation and behavioural control.
• Pavlovian, habitual and goal-directed behaviours explained with one scheme.
• A possible phylogenetic trajectory from simpler to hierarchical controllers.
• Precision-dependent processes regulate habitual and goal-directed behaviour.

## 1 Introduction

*The animal must respond to changes in the environment in such a manner that its responsive activity is directed towards the preservation of its existence. This conclusion holds also if we consider the living organism in terms of physical and chemical science. Every material system can exist as an entity only so long as its internal forces, attraction, cohesion, etc., balance the external forces acting upon it. […] Being a definite circumscribed material system, it can only continue to exist so long as it is in continuous equilibrium with the forces external to it*. **Ivan Pavlov**

Current associative learning theories in psychology and neuroscience assume that animal behaviour depends on multiple forms of control (i.e., Pavlovian, goal-directed, and habitual processes). These control schemes are based on associations between stimuli, actions and outcomes and are either innate or learned through experience.

Our aim is to offer an integrative perspective by contextualizing classical formulations of adaptive behaviour within the Active Inference framework, which extends predictive coding from the domain of perception to cover action (Friston et al., 2009). Active Inference assumes that organisms act to fulfil prior expectations that encode the (evolutionarily) values of their states (e.g., having access to food). The mathematical foundation of Active Inference rests on the notion of *free energy minimization*, where the long-term average of free energy approximates the entropy of sensory states. Minimizing free energy (and therefore entropy) enables an organism to resist the dispersive effects of “*external forces acting upon it*” to ensure “*it is in continuous equilibrium with the forces external to it*” (Pavlov, 2010). Crucially, free energy can also be interpreted in a statistical sense as an approximation to Bayesian model evidence. This means Active Inference can be described normatively as maximizing (a negative free energy bound on the logarithm of) Bayesian model evidence. In other words, minimizing free energy reduces the discrepancy (e.g., prediction error) between sensations and their predictions. This discrepancy can be reduced by changing predictions – through perception – or by selectively sampling sensory inputs that were predicted – through action (Friston, 2010).

The basic premise of this article is that the ontology of behavioural paradigms in associative learning can be seen as a successive contextualisation of more elemental sensorimotor constructs, within generative models of increasing hierarchical depth. This formulation explains how the primitive sensorimotor architecture of homeostatic control – of our early evolutionary ancestors – evolved towards goal-directed and prospective forms of control. This phylogenetic progression rests on the hierarchical elaboration of more primitive architectures (Cisek and Kalaska, 2010, Pezzulo and Castelfranchi, 2009). Furthermore, this hierarchical elaboration dissolves any apparent mechanistic distinction between the optimization processes that underlie different control or learning schemes, suggesting that they are all manifestations of Active Inference – under various contexts or conditions (Friston et al., 2009). This novel hypothesis contrasts with the standard (associative learning) view that the computations underlying different forms of behavioural control are fundamentally different and appeal to different (optimization) principles.

We first review associative learning theories of homeostatic and behavioural control. We then offer an Active Inference formulation of adaptive behaviour that fulfils homoeostatic imperatives in increasingly sophisticated ways – building upon peripheral (somatic and autonomic) reflexes to explain simple Pavlovian and instrumental motor responses and, finally, complex goal-directed behaviour. A crucial aspect of this hierarchical perspective is that higher-level hierarchical representations contextualize lower levels and predict longer sequences of cues and responses. This is accommodated by predictions about transitions over increasingly protracted time scales (Friston, 2008, Pezzulo, 2012).

## 2 Homeostatic regulation and adaptive behavioural control in associative learning theories

Cannon (1929) first proposed that the evolutionary function of physiology and behaviour is to restrict homeostatic states to a physiologically tenable range. Homeostatic regulation therefore allows animals to maintain a “*continuous equilibrium*” between the internal milieu and environmental states, which (Pavlov, 2010) considered the *raison d’être* for our brains.

The regulation of homeostatic states – or of allostatic processes (Sterling and Eyer, 1988) – has long been described in terms of control-theoretic and cybernetic mechanisms of error cancellation and feedback control (Ashby, 1947). At the neurobiological level, one hypothesis is that homeostatic control requires *interoceptive signals* that report current homeostatic levels (e.g., the current level of glucose in the blood) (Craig, 2010, Damasio and Carvalho, 2013, Gu et al., 2013). A hungry animal can be described as an animal whose homeostatic condition departs significantly from a level that is ‘good’ for survival. With some simplifications, the ‘good’ level of glucose is used as a reference for the controller to steer action (e.g., ingest food to restore the level of glucose).

This form of autonomic regulation involves triggering autonomic reflexes that control bodily processes such as heart rate, blood pressure and peristalsis. Under some conditions, autonomic reflexes can restore homeostatic levels (e.g., a hyperthermic animal can cool down by perspiring). Although elemental, autonomic reflexes are not sufficient to fully support homoeostasis: to satisfy hunger or thirst, the animal must act on the external world. Early theories of homeostatic regulation focused on simple (e.g., approach or avoidance) constituents of an innate behavioural repertoire. However, higher animals learn to achieve their goals in complex and flexible ways that go well beyond approach and avoidance. To do this they must acquire an adequate behavioural repertoire and learn to select from currently available actions or sequences of action (policies). This is the main focus of associative learning theories in psychology and biology.

### 2.1 A taxonomy of behavioural controllers in the associative learning literature

Contemporary associative learning theories assume that action selection depends on the continuous cooperation and competition of several behavioural controllers, which can be divided into “Pavlovian” and “instrumental” (goal-directed and habitual) (Balleine and Dickinson, 1998, Daw et al., 2005, Dayan, 2009).

*Behavioural reflexes* represent a basic form of controller that constitutes the innate repertoire of most animals. This controller is rather limited, as it calls on a limited set of *unconditioned responses* (e.g., approaching and ingesting food or withdrawing from a painful stimulus) in response to a circumscribed and predefined class of stimuli (called *unconditioned stimuli*). Still, this controller is sufficient for most animals to survive, even without any experience-dependent learning.

*Pavlovian (classical) conditioning* is the process by which an unconditioned stimulus (say, food), that triggers an unconditioned reflex (say, salivation), is repeatedly paired with a neutral stimulus (say, a bell). The pairing is thought to produce stimulus-stimulus associations (between the food and the bell). After several pairings, the (formerly) neutral stimulus is able – on its own – to trigger a reflex called a *conditioned response* (salivation when the bell rings). An evolutionary imperative for acquiring a conditioned response is that the stimulus-stimulus associations capture (ecologically) valid statistical contingencies: if conditioned stimuli are good predictors of unconditioned stimuli, they have inherent relevance for survival. Classical conditioning mechanisms generally have limited scope and only capture a small range of predefined responses – not the acquisition of novel behaviours.

More complex forms of associative learning guide the acquisition of instrumental responses, which are assumed to maximize some goal or value function. Among these, flexible forms of control consider *goal-directed actions*. For example, while ingesting food can be considered as a behavioural reflex for a hungry animal, foraging for food is a complex skill – requiring the acquisition and coordination of action sequences over time. Truly goal-directed actions are considered to be oriented towards (and engaged by) a goal (i.e., an action outcome, say the expected sound of a ringing bell or the predicted sight or taste of a food).

Empirically, a behaviour is considered to be goal-directed if, and only if, it is sensitive to changes in reward contingencies, as revealed by reward devaluation and contingency degradation paradigms (Balleine and Dickinson, 1998). This sensitivity gives goal-directed actions their characteristic flexibility and context sensitivity. However, this comes at a cost: to act in a goal-directed manner an animal must know (learn) the causal effects of its actions. This process is often described as the acquisition of sets of associations (Dickinson, 2012), including (bidirectional) action-outcome associations. When used in the forward direction (from action to outcome), these associations permit the prediction of the sensory consequences of possible actions. When used in the backward direction (from outcome to action), the implicit mapping enables the selection of an action that produces the (desired) consequences. This computational perspective maps nicely onto *ideomotor theory* (Hommel et al., 2001, Prinz, 1997) and the distinction between *forward* and *inverse models* of motor control (Wolpert et al., 1998). Note that all these computations must be contextualized, because any given action can produce different outcomes in different situations. This implies that the selection of a specific goal-directed action requires a complex computational process (formally, the solution to an *inverse problem*). In this sense, action selection depends on the sort of deliberation found in model-based schemes, such as tree searches (e.g., used to solve Markov decision problems) or ‘planning-as-inference’ (Botvinick and Toussaint, 2012) – the latter being closely related to Active Inference (see below).

A simpler kind of instrumental controller involves routines or *habits*. Habits do not require purposeful deliberation and are stimulus driven (not goal-driven): a stimulus activates simpler “cached” action-value representations, learned through experiencing past reinforcements. This renders habitual computation quite ‘cheap’, but at the expense of flexibility. For example, habitual mechanisms do not adapt immediately to changes in reward contingencies but need extensive re-learning.

The controllers considered above interact by cooperating or competing. For example, it has been suggested that habitual and goal-directed control compete to control action based on uncertainty (Daw et al., 2005). In addition, various bidirectional interactions between instrumental and Pavlovian mechanisms have been proposed (Dayan, 2009). More recent formulations emphasize cooperation, rather than only competition (Daw and Dayan, 2014, Dolan and Dayan, 2013, Keramati et al., 2011, O’Doherty et al., 2015, Pezzulo et al., 2013; see also Daw et al., 2011, Lee et al., 2014) for relevant empirical evidence that supports an integrative viewpoint. In particular, these accounts are consistent with the current view in that it is the precision or reliability of alternative controllers that arbitrates their relative contribution. These treatments rest on normative principles and explore various trade-offs between (mental or fictive) exploration and exploitation; for example, trading fast inaccurate solutions for slow accurate solutions. However, these formulations tend to assume that different controllers conform to distinct computational principles (say, model-based vs. model-free) and – with some noteworthy exceptions (Dezfouli and Balleine, 2012, Pezzulo and Castelfranchi, 2009) – do not consider how more complex controllers could have developed from earlier (less flexible) controllers.

Below, we introduce a view of adaptive behaviour in which behavioural controllers are assimilated successively into an Active Inference scheme; showing how more complex forms of control are elaborated from simpler processes – thereby contextualizing and linking them hierarchically in terms of their temporal and functional scope.

## 3 Homeostatic processes and adaptive behavioural controllers in Active Inference

Active Inference assumes that the brain is a statistical organ that learns a generative model of its environment (Dayan et al., 1995, Friston, 2010, Friston et al., 2009, Helmholtz, 1866). The theory extends predictive coding to the domain of action-perception loops. In predictive coding, perception is regarded as an inference process (Gregory, 1980), whose aim is to minimize prediction errors or the difference between empirical priors (which play the role of perceptual hypotheses) and current sensations. *Empirical* priors are just representations or expectations that provide top-down predictions in hierarchical models of how sensations are generated. Prediction errors at every level of the hierarchy are then minimized by adjusting (empirical) prior expectations. This optimization process can be understood, neurobiologically, as recurrent message passing among levels of the sensorimotor hierarchy. The resulting optimization of prior expectations or beliefs corresponds to perceptual inference. Crucially, Active Inference considers another way to minimize prediction errors; namely, through action.

**Fig. 1:**
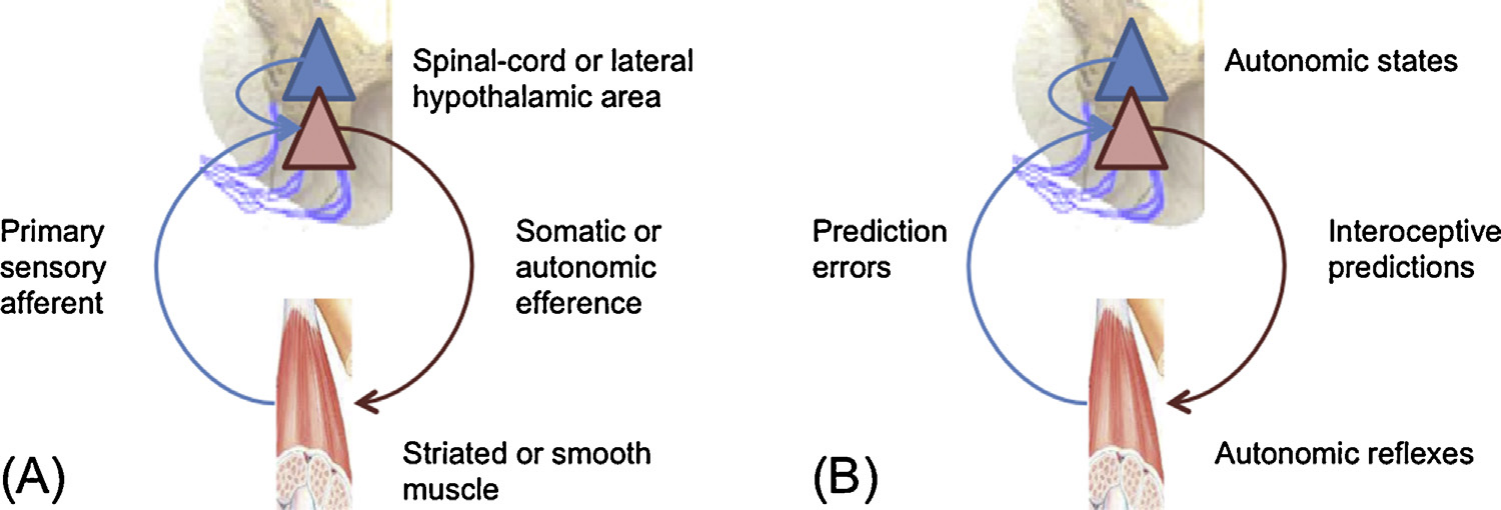
Peripheral reflexes in Active Inference. Left: the general structure of a (close) control loop, which applies to both the anatomy of classical motor arc reflexes and autonomic reflexes. Right: Active Inference view of autonomic regulation (using the same scheme as the left graphic). Interoceptive prediction errors (e.g., the difference between the expected level of glucose and the currently sensed level) can be suppressed by autonomic reflexes, much like proprioceptive prediction errors (e.g., the difference between the expected and actual position of my finger) can be suppressed directly via arc reflexes.

The simplest form of action corresponds to *peripheral reflexes*, which can be of an autonomic or somatic sort – depending upon whether the muscles engaged are smooth or striated: see Fig. 1 (left). From the point of view of Active Inference, the purpose of a peripheral reflex is to suppress proprioceptive or interoceptive prediction errors through closed loop control (Friston, 2011). The *set point* or *equilibrium point* of the reflex can be nuanced by descending predictions that project to prediction error populations (red triangles) that send efferents to neuromuscular junctions. This efferent outflow will cease when the interoceptive or proprioceptive feedback matches the descending prediction (blue arrows).

### 3.1 Homeostatic regulation through autonomic reflexes and unconditioned responses

The aforementioned control loop is usually associated with arc reflexes and the suppression of *proprioceptive* prediction errors through action (Feldman and Friston, 2010, Shipp et al., 2013). In addition, the same loop can perform homeostatic regulation through *autonomic reflexes* (Pezzulo, 2013, Seth, 2013, Seth et al., 2012) – a process that has long been understood in terms of error cancellation (Cannon, 1929). The Active Inference loop shown in Fig. 1 (right panel) illustrates how autonomic reflexes minimize interoceptive predictions and thus assure ‘good’ levels of autonomic states. This process introduces a *circular causality* between homeostatic variables and autonomic reflexes, as the former triggers the latter and vice versa. Autonomic reflexes can also facilitate other homeostatic reflexes, as illustrated in Ivan Pavlov's example of how salivation facilitates ingestion: “Edible substances evoke the secretion of thick, concentrated saliva. Why? The answer, obviously, is that this enables the mass of food to pass smoothly through the tube leading from the mouth into the stomach.” (Ivan Pavlov, 1904 Nobel Lecture).

Although autonomic reflexes allow a continuous monitoring and control of bodily events, they are not sufficient for allostasis in complex environments – animals must take action to survive. Ultimately, actions must be selected that enable them to maintain some states (e.g., glucose level) within a viable range; either directly (homoeostasis) or indirectly (allostasis). However, allostatic control is a difficult control problem, as there is a necessarily *distal temporal relation* between actions and their consequences, particularly at the level of homeostatic regulation and interoception. For example, even if I act now (e.g., I eat or go foraging), the level of glucose in my blood changes minutes or even hours later. This so-called *credit assignment* problem makes closed loop homoeostatic control of interoceptive prediction errors problematic. How then should one choose the appropriate sequence of actions to maintain homeostasis?

Many researchers have proposed that the solution to this problem rests on re-representing bodily (and related) events *centrally*, through various *interoceptive* channels (Craig, 2010) – and also in the form of *feelings* that influence decision-making (Damasio and Carvalho, 2013). In this vein, we suggest that the best way to finesse the credit assignment problem is to learn (hierarchical) models of how interoceptive signals are generated. Much like perceptual hierarchies, these *interoceptive models* predict interoceptive events over different timescales and thus anticipate homeostatic needs.

Homoeostasis is often described as a reactive mechanism but in fact it can be more sophisticated than that (Barrett and Simmons, 2015). Animals do not actually react to hypoglycaemic sensations, but much earlier, so that they can act (e.g., eat) before dangerous and unpredicted internal states (e.g., hypoglycaemia) are experienced. This anticipatory ability can be supported by generative models describing how interoceptive signals change over time, conditioned on some (future) behaviour. For example, prior to an energy-consuming activity, one can anticipate its hypoglycaemic consequences and prepare food. Many homeostatic phenomena are periodic and this makes their prediction possible, at least to some extent. Further, evidence indicates that hormones linked to metabolic processes such as insulin (Woods et al., 1970) and ghrelin (Drazen et al., 2006) can be conditioned and initiate anticipatory allostatic processes. By analogy, using interoceptive prediction errors to infer a preparatory behavioural set – before homoeostatic extremis (e.g., profound hypoglycaemia) – allows animals to act in advance and, in essence, avoid *anticipated* losses of glucose rather than react to actual losses. Conceptually, this speaks to the tenet of the good regulator theorem; namely, that any allostatic or homoeostatic system must entail a model of its exchange with the environment (Conant and Ashby, 1970).

**Fig. 2:**
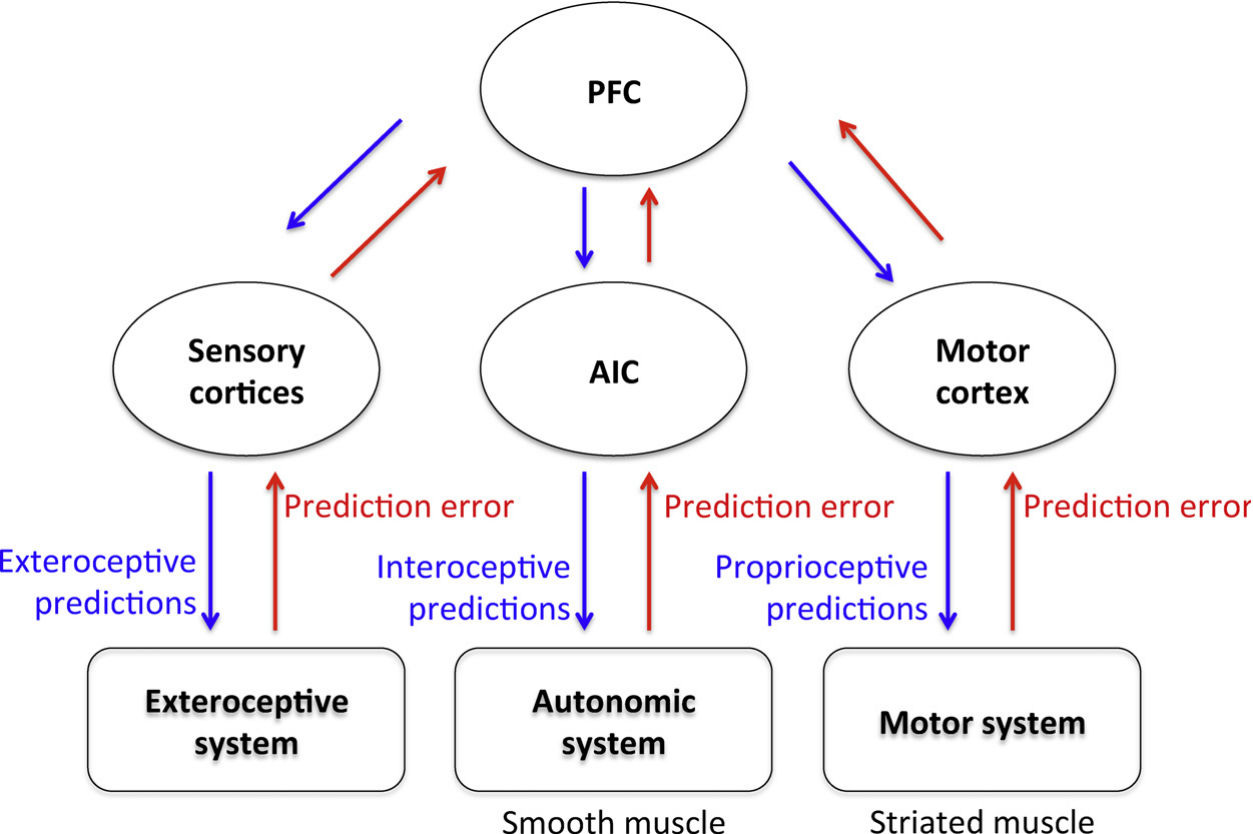
Exteroceptive, interoceptive, and proprioceptive channels are partially separated in the brain. In this schematic, exteroceptive, interoceptive and proprioceptive systems are shown as being relatively separated in terms of hierarchical neuronal systems. At the point of convergence – at higher levels of the hierarchy (here the Prefrontal Cortex) – the representations become amodal or multimodal – providing descending predictions in the exteroceptive, autonomic and proprioceptive domains. These descending predictions engage autonomic and motor reflexes by resetting their fixed points while, at the same time, being informed by ascending prediction errors. AIC: anterior insular cortex.

Another pointer towards a hierarchical architecture is that – in the brain – interoceptive and proprioceptive channels are not directly interconnected at lower hierarchical levels; see Fig. 2. While autonomic reflexes can directly minimize interoceptive prediction errors through reciprocal message passing, arc reflexes can only be engaged vicariously through higher (multimodal) cortical and subcortical systems. The homeostatic variables that have to be maintained within a safe range cannot be (technically speaking) *controlled* directly. In other words, it is not possible to use interoceptive prediction errors directly to enslave action in the same way as proprioceptive prediction errors produce movement. However, at a slower timescale, higher levels – that integrate multimodal information including ascending interoceptive prediction errors about impending homoeostatic violations – are in a position to engage action through descending proprioceptive predictions (see below).

In Active Inference, these central representations of bodily events take the form of hierarchical *generative* models – models that describe the consequences of sensorimotor contingencies at multiple (exteroceptive, proprioceptive, and interoceptive) levels. The models are called ‘generative’ because they can generate – in a top-down manner – the expected (sensory) consequences of *latent* (hidden) causes.

The generative model shown in Fig. 2 links exteroceptive, proprioceptive, and interoceptive information by jointly representing their hidden or latent causes (e.g., a cause can embody the prior knowledge that the sight of a burger will produce certain visual and gustatory sensations, that it affords a grasping action, and ultimately that it increases the level of glucose in the blood). The same idea arises in *embodied predictive coding*, where perceptual inference minimizes both sensory and proprioceptive error (Pezzulo, 2013). These models necessarily entail a *sensorimotor-to-interoceptive* mapping that encodes associations between sensorimotor and interoceptive events; say, “a burger in my mouth” (a sensorimotor event) generates (and predicts) “more glucose in my blood” (an interoceptive event). The acquisition of these models is made possible by the fact that sensorimotor events are reflected by variations in the interoceptive domain, as the two are causally related (e.g., through the embodied gastric system). The lowest levels of these models might be reified in anatomy; for example, embodied in the functioning of the gastric system (Mayer, 2011).

In sum, generative models linking sensorimotor and interoceptive events resolve a key aspect of homeostatic regulation. They enable the use (and suppression) of sensorimotor prediction errors as *proxies* for (the suppression of) interoceptive prediction errors, which are hard to control at short timescales. For example, I can harness the association between ingesting a burger, the sensation of a full stomach and the (future) restoration of blood glucose levels. These associations mean that the exteroceptive prediction errors (that report the fact that I am not currently eating) can be resolved by acting (eating) to suppress interoceptive prediction errors *in the future.* In this view, while the variable to be *controlled* is homeostatic, the states that an organism *controls* are sensorimotor (e.g., eating). This perspective also speaks to valence and feelings – and their putative adaptive functions (Damasio and Carvalho, 2013).

**Fig. 3:**
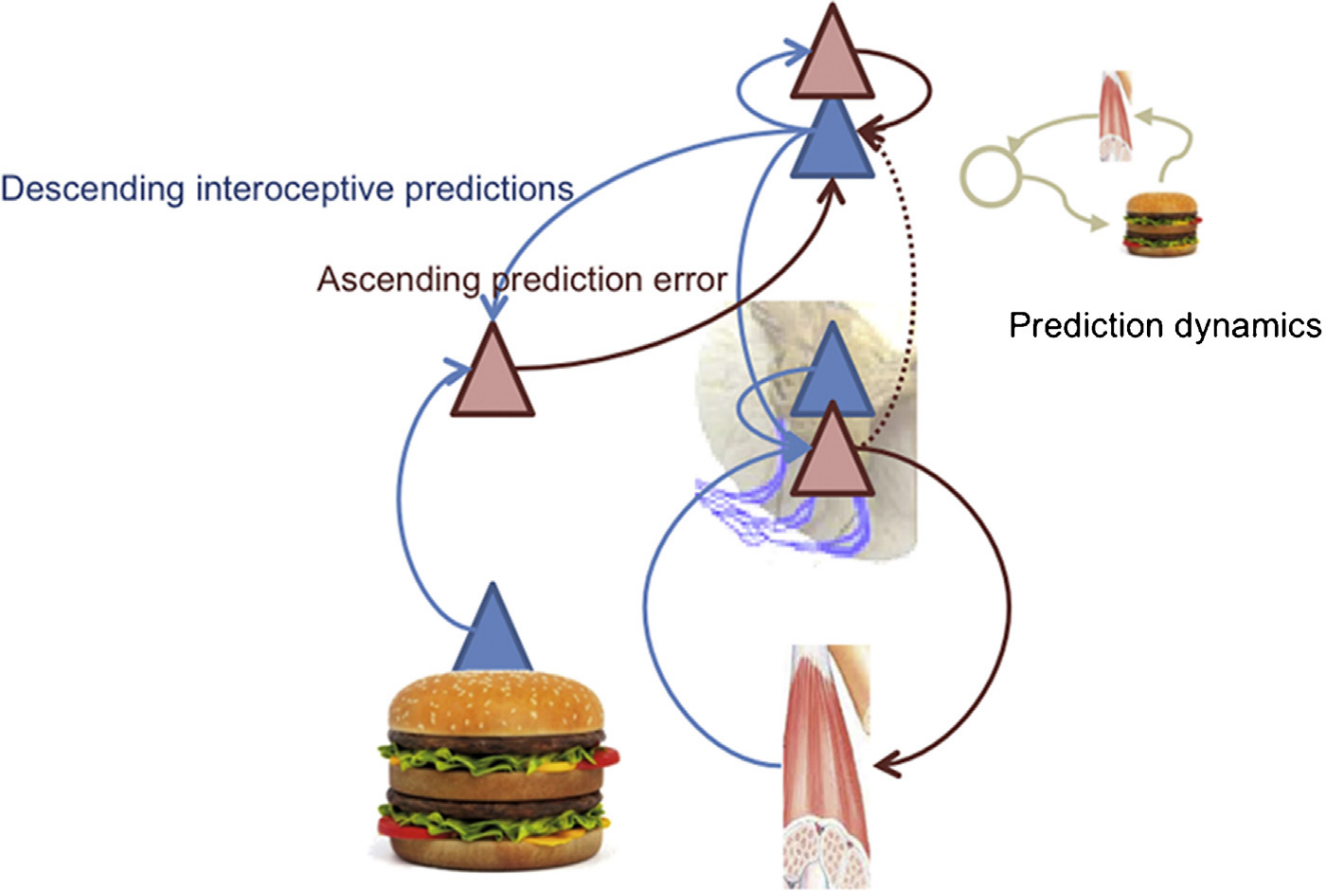
Unconditioned responses. Dynamical control over the equilibrium point of peripheral reflexes rests on descending predictions about proprioceptive or interoceptive input. Peripheral reflexes are now contingent upon top-down predictions that are themselves informed by ascending prediction errors from – in this example – gustatory input. The timing of the various cues may or may not be important. In this cartoon, the temporal contingencies are modelled through interactions between the error (red) and expectation (blue) populations at the top of the hierarchy. In dynamical schemes, these interactions produce an attractor or heteroclinic cycle that models the temporal succession of predicted sensations (here, gustatory and subsequent interoceptive sensations).

Fig. 3 shows an Active Inference formulation of unconditioned responses. This is an extension of the reflexive control loop of Fig. 1 that places the peripheral reflexes under higher (central) control, through the provision of descending predictions that provide a reference point or set point. This architecture bridges the gap between interoceptive signals (e.g., a gustatory input) and autonomic responses (e.g., salivation). The (unpredicted) gustatory sensations of food can elicit ascending gustatory prediction errors that in turn elicit descending predictions of taste sensations and (consummatory) interoception. The descending interoceptive predictions drive autonomic reflexes – mediating a stimulus-response mapping. However, this mapping is established through the vertical integration of predictions and prediction errors, in the sense of hierarchical inference or predictive coding. This fact marks an important difference with the associative learning literature, where unconditioned responses are supposed to be controlled by cues (i.e., they are stimulus-response mappings). In Active Inference, they are implemented within generative architectures that encode stimulus-stimulus associations or contingencies, where responses are elicited peripherally to minimize (interoceptive or proprioceptive) prediction error. In this setting, a prediction encoded at higher hierarchical levels (say, the sensation of tasting a burger) becomes an equilibrium or ‘reference point’ in control-theoretic formulations.

Besides their importance per se, unconditioned responses are crucial to scaffold increasingly more complex forms of behavioural control that are acquired through learning (rather than being innate). These new strategies essentially extend and contextualize the animal's innate repertoire of stimulus bound responses in an open-ended way.

### 3.2 Towards more sophisticated forms homeostatic regulation: the hierarchical architecture supporting adaptive behavioural control

Animals can come pre-equipped with a fixed repertoire of reflexes (e.g., approaching-food behaviour) enabling them to satisfy their basic needs (e.g., hunger), under restricted conditions. However, they can learn increasingly more sophisticated ways to maintain homeostasis (e.g., from eating a food in front of me, to opening a fridge, to buying food for dinner).

Associative learning theory describes these strategies in terms of multiple behavioural controllers (i.e., Pavlovian, goal-directed and habitual). In Active Inference, these forms of control are not segregated, but it is possible to subsume them within a hierarchical architecture that builds on the control loop shown in Fig. 1, Fig. 3. High levels in hierarchical models contextualize lower level expectations through descending or backward connections, and eventually, at the peripheral level of the hierarchy, nuance motor and autonomic reflexes (which are thus conserved in an evolutionary sense). In this scheme, a cascade of descending predictions set the equilibrium points of reflexes, where these predictions are – in principle – informed by deep hierarchical processing.

We now review this formulation, beginning with a focus on what associative learning theories call *control*, namely how behaviour arises from different processes; and then considering how these processes are *learned* in the face of new experience (Mackintosh, 1983).

#### 3.2.1 Pavlovian responses

**Fig. 4:**
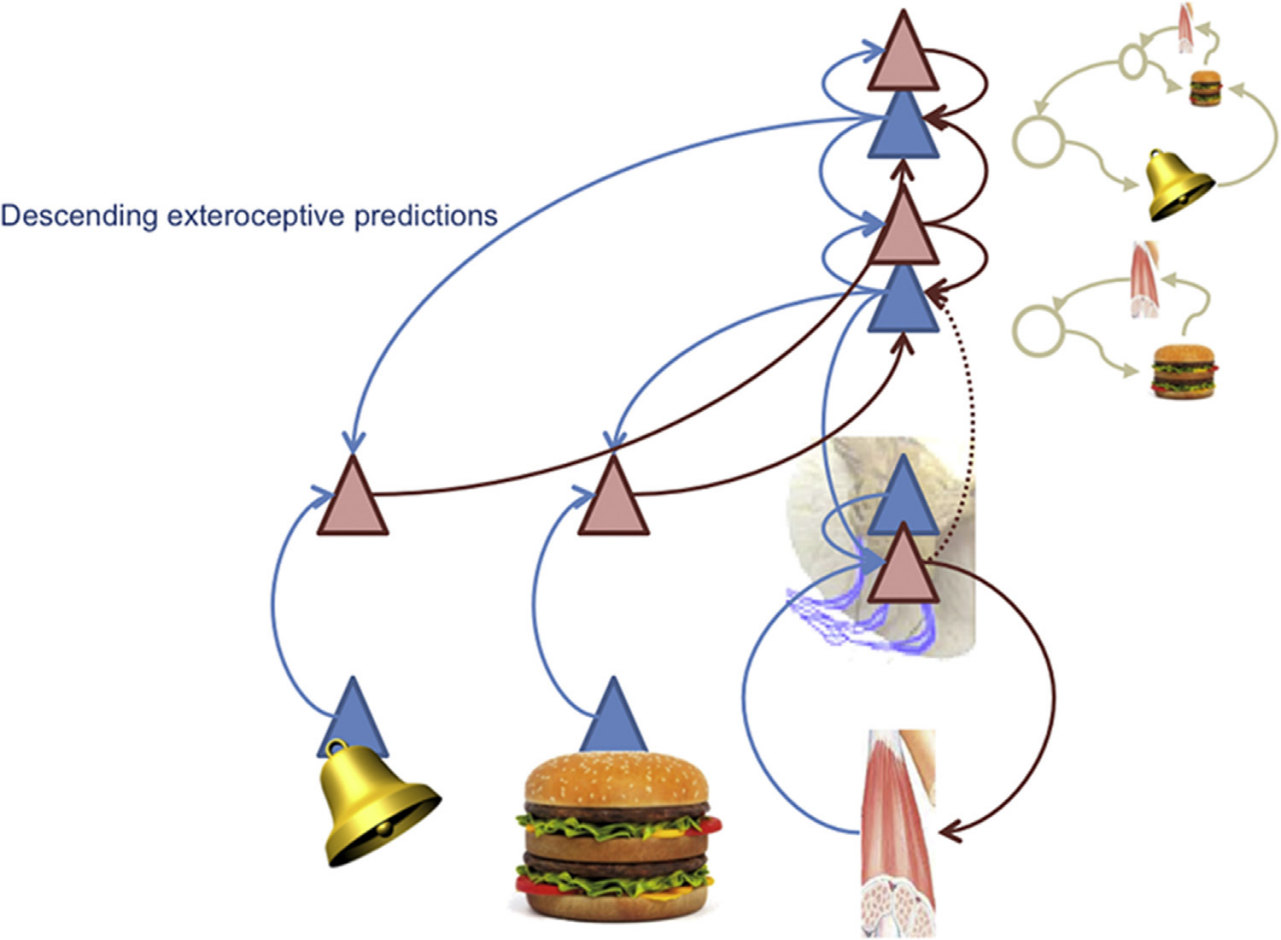
Pavlovian responses. In this schematic, we have added a hierarchical level that incorporates exteroceptive predictions (here, of a ringing bell) into its model of state transitions. This higher-level construct represents the sound of a bell that portends interoceptive changes that induce autonomic reflexes (or unconditioned responses). Because the highest level now provides predictions in the exteroceptive and interoceptive domain, it enables perceptual inference to elicit autonomic responses, providing interoceptive predictions that can be fulfilled by smooth muscle reflexes. Here, the agent's model of the world involves a bell ringing that causes gustatory events that induce salivation. Optimizing expectations about auditory objects corresponds to predictive coding of auditory input and the perception of bell ringing; while descending interoceptive predictions cancel gustatory prediction error and descending predictions about the state of smooth muscle reflexively elicit salivation.

Based on the central (sensorimotor) predictions in Fig. 3, Pavlovian control arises when descending predictions encompass predictive exteroceptive cues. In this case, the model acquires *exteroceptive*-to-*interoceptive* sensory mappings, where the first sensory event is the conditioned stimulus (say a bell) and the second is an unconditioned stimulus (say, the taste of a burger). As shown in Fig. 4, the *stimulus-stimulus* associations underlying Pavlovian conditioning are instantiated by a high-level representation of a conditioned stimulus that is inserted into the sequence of expected events. This means that the conditioned stimulus (here the sound of a bell) elicits auditory prediction errors that engage top-down predictions of appropriate auditory input, which – at the same time – predict the emergence of interoceptive sensations that produce the unconditioned reflex. Descending predictions engage peripheral reflexes – establishing a (vertical) link between the conditioned stimulus and unconditioned response. Here, the highest-level expectations absorb the unconditioned reflex into dynamical predictions that support Pavlovian responses.

Note that in this scheme, different hierarchical levels of the generative model act in concert. The aforementioned closed loop reflexes afford a basic (but restricted) repertoire of responses to salient sensory events. Sensory-to-sensory associations expand this initial repertoire beyond cues that are specified innately. The hierarchical composition of models permits the open-ended evaluation of sensory events and adaptation to changing environmental contingencies (which would be precluded by purely innate mechanisms). Finally, as we will see below, incorporating proprioceptive predictions enables conditioned stimuli to garner instrumental responses through classical motor reflexes.

It is worth noting that associative explanations of conditioning – based on the pairing of (conditioned and unconditioned) stimuli – have proven to be somewhat limited; for example, they do not cover associations between stimuli and context. Rather, it has been proposed that conditioning depends on expectations based on knowledge of the relations between events at large (Rescorla, 1988). In terms of Active Inference, this implies that the generative models not only encode associations between stimuli or between conditioned stimuli and reward delivery; rather, they are *latent cause models* that reconstruct the hidden processes jointly responsible for context, sensory and reinforcing events (Courville et al., 2006). Note that latent cause models have not been generalized to instrumental responses – but this generalization is straightforward in Active Inference, because the predictions generated by the models can elicit action.

#### 3.2.2 Instrumental responses

A further hierarchical extension now allows the addition of proprioceptive and exteroceptive cues that lead to the Pavlovian response. This provides a mechanistic basis for *instrumental* behaviour, which is usually linked to habitual control in associative learning models and permits adaptive behavioural patterns to be stabilized through learning.

**Fig. 5:**
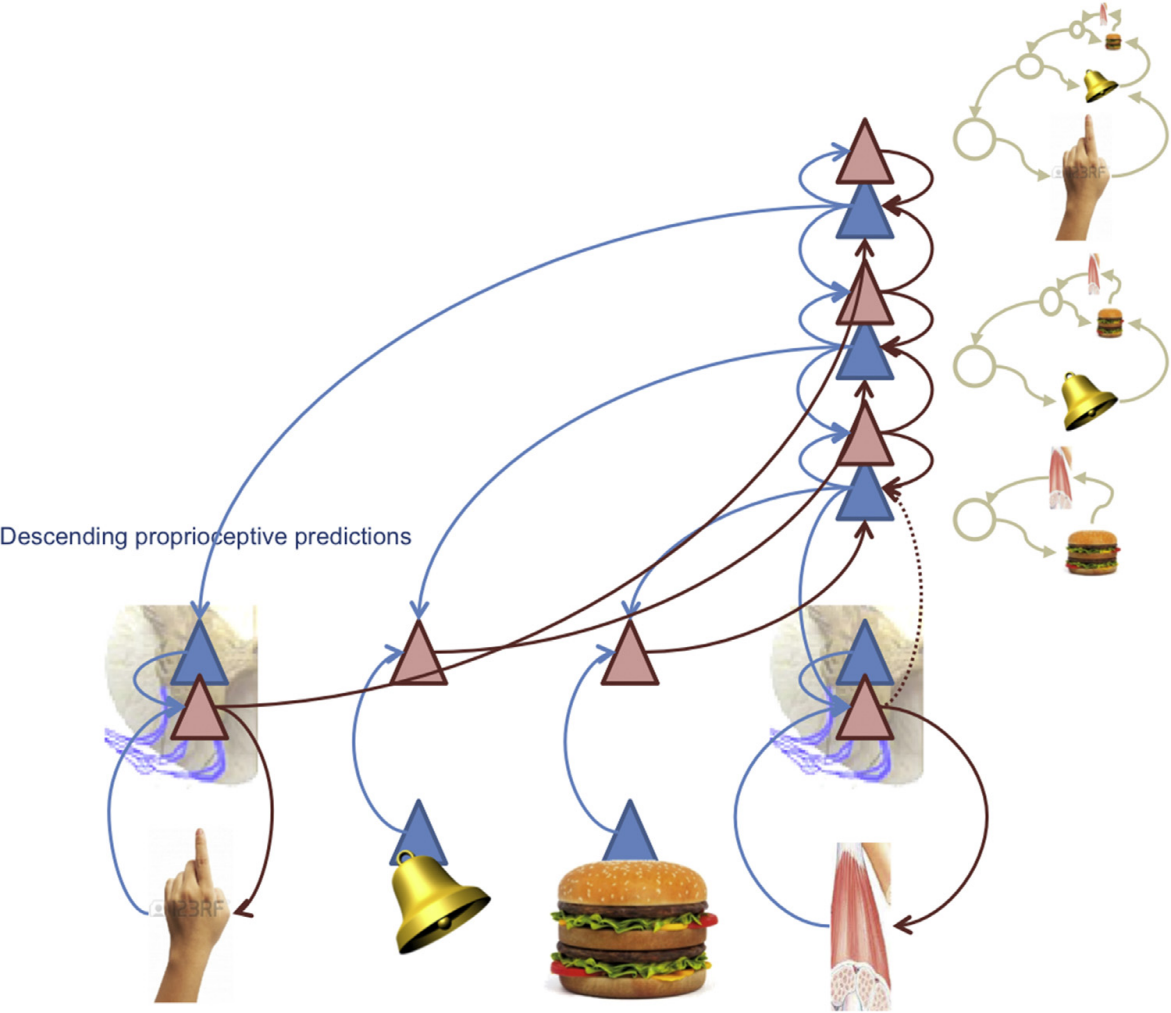
Instrumental responses. Here, we have supplemented the hierarchy of Fig. 6 to include proprioceptive precedents of a conditioned exteroceptive stimulus – here some hand movement. This means that moving the hand induces expectations or predictions about the sound of the bell that, in turn, elicit lower-level expectations leading to a conditioned response. In other words, this hierarchical extension enables Pavlovian conditioning to enslave instrumental responses.

In the example shown in Fig. 5, imagine that an animal has learned that a bell rings whenever its hand moves. This association is encoded by high-level representations of the implicit conjunction of proprioceptive and auditory input that plays the role of a sensorimotor construct or contingency (O’Regan and Noe, 2001). Crucially, the motor responses producing the predicted proprioceptive input will be elicited reflexively (through peripheral reflexes), whenever they are engaged by descending predictions.

Note how the sound of a bell engenders posterior expectations about the Pavlovian response that – mediated by prediction errors ascending to the top of the hierarchy – will produce posterior expectations about the conditioned response. This may be a plausible mechanism for Pavlovian-Instrumental transfer (PIT) and in particular PIT-specific phenomena (Corbit and Balleine, 2011).

#### 3.2.3 Goal-directed behaviour

**Fig. 6:**
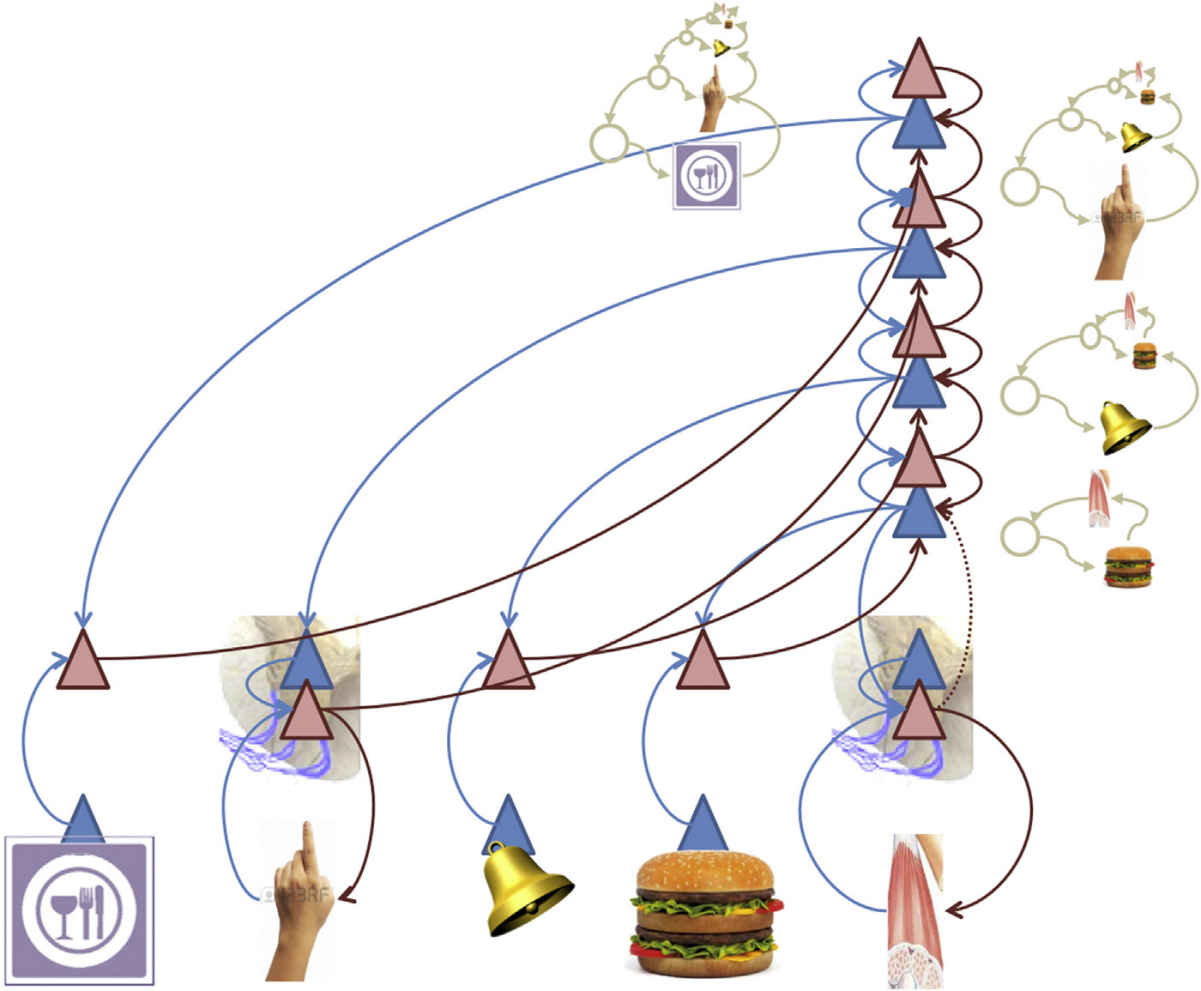
Goal-directed responses. In this final extension, we have added contextual exteroceptive cues (e.g., a restaurant sign) that enable the highest level to select the sensorimotor contingencies underlying instrumental or conditioned responses. Goal-directed behaviour can be regarded as the highest level of (model-based) control that embodies all the ingredients of Active Inference.

The aforementioned scheme can be equipped with a further hierarchical extension that predicts contextual cues associated with instrumental responses. Fig. 6 illustrates a simple example of goal-directed behaviour – choosing somewhere to eat – which extends the previous example using conditional or contextualizing (exteroceptive) stimuli; e.g. a restaurant sign. In this example, descending predictions select the conditional response through adjusting the *gain* or *precision* of prediction errors at the lower instrumental level in the hierarchy (circular arrowhead). In Active Inference, a high precision implies that descending predictions will dominate posterior beliefs. This ensures that the predicted sequence of behaviour is enacted, through peripheral reflexes (Friston et al., 2012a). Later, we will consider the role of dopamine in optimizing the precision of action selection in this way.

The key distinguishing feature of goal-directed systems rests on the form of the inference they require. In contrast with Pavlovian and instrumental controllers, goal-directed systems represent (counterfactual) future states, and minimize the difference between the preferred or goal state and outcomes predicted from the current state. See Friston et al. (2013) for a formal example using Markov decision processes. This prospective form of control is supported by the ability of higher hierarchical levels to anticipate the future and to select policies that enslave action (Friston et al., 2010, Pezzulo, 2012).

In contradistinction to classical controllers, the generative models described above capture increasingly more distal relations between actions and outcomes (say choosing food from a menu or even buying food to put in the fridge) that can be encoded in the form of extended cue-response sequences in higher hierarchical levels (Friston, 2008). They thus enable the modelling of *counterfactual* outcomes and the selection of policies that allow goals to be reached. Furthermore, these models can accommodate generic priors, such as action costs. However, the prosecution of control sequences still calls on exactly the same hierarchical structures that underpin more elemental and unsupervised forms of learning – right down to motor and autonomic reflexes in the periphery.

#### 3.2.4 The interaction between controllers in the Active Inference framework

Associative learning theories hypothesize that behaviour results from the interaction between different controllers. For instance – in relation to goal-directed and habitual interactions – an influential model proposes that the two controllers compete to control behaviour on the basis of their relative precision or confidence (Daw et al., 2005). In Active Inference, the apparently distinct forms of control map onto specific levels within a single hierarchical architecture. Indeed, the different controllers can be represented along a continuum from sophisticated goal-directed models (Bayesian graphs) to simpler sensorimotor loops or subgraphs. Higher order levels contextualize lower levels through backward connections that provide empirical priors. Crucially, these descending expectations optimize both content and context – in the form of *expected states* and *expected precision*, respectively. The descending influence of higher order levels depends on the relative precision (or confidence) of prediction errors at each level of the hierarchy. When higher levels have greater precision, their contextual influence dominates; whereas, when expected sensory precision is high, inference and subsequent behaviour is driven by sensory evidence. This fits comfortably with the relative influence of hierarchically deep (goal directed) and shallow (habitual) levels of control that are balanced in terms of their relative salience or, in predictive coding, precision (FitzGerald et al., 2014).

In Active Inference, control is not dichotomized into two discrete systems, but viewed as distributed along a graded continuum going from the highest levels of abstract, prospective and conscious reasoning to more concrete, short-sighted unconscious levels of reasoning down to the arc reflex. See Bates and Elman (1993), for similar considerations in the context of connectionist models. Within this framework, phenomena usually interpreted as arising from conflict between different controllers (e.g., goal-directed vs. habitual) can be reinterpreted as the failure of high-order levels to contextualize lower-order levels. For example, devaluation occurs when an animal – after learning an action-outcome contingency (e.g., lever pressing to obtain food pellets) – stops performing the action after the outcome associated with the action has lost is palatability (e.g., after satiation). A large body of evidence has shown that overtrained, contrary to undertrained, rats are unaffected by devaluation, since they continue to perform the action even when the associated outcome ceases to be palatable (Balleine and Dickinson, 1998). Current associative learning theories interpret the resistance to devaluation in overtrained rats as a replacement of goal-directed control – based on action-outcome contingencies – by habitual control – based on stimulus-reward learning.

From an Active Inference perspective, a resistance to devaluation can be interpreted as a failure to contextualize low-level instrumental inference (including sequences of predictions about the proprioceptive sensations associated with the action performance; e.g., those associated with lever pressing and food pellets consumption) by higher levels that infer the motivational state. This failure is due to the fact that the low level, after over-training, has acquired a relatively higher precision and is therefore unaffected by descending predictions that have access to motivational information. Although higher levels may ‘know’ that performing the action will not lead to a valuable outcome, they have lost the ability to attenuate the precision of sensory levels during overtraining (where the animal has learned this attenuation is redundant). This can be modelled in the Active Inference framework by placing a high precision on sensory prediction errors, which can then activate reflexes directly.

More generally, placing a high precision on sensory prediction errors produces habitisation (i.e., the shift from goal-directed to habitual control), where habits directly activate reflexes and preclude (unnecessary) inference at higher hierarchical levels. This produces the equivalent of a so-called habitual controller, which does not depend on goals encoded at higher hierarchical levels but rather acts reactively. In other words, a stimulus (say, the visual impression of a lever) triggers descending predictions of lever pressing sensations, which (when afforded high precision) engages an arc reflex. Lever pressing then minimizes (precise) sensory prediction error, even when the further consequences of pressing a lever, say receiving food, are neither predicted nor desired. Habitisation can help saving (cognitive or other) resources, but it could also be involved in some forms of addiction. For example, an initially outcome-based drug taking behaviour can become insensitive to the actual effects of the drugs, and be controlled directly by a drug-predicting cue (Robbins and Everitt, 1999). Habitisation can occur at multiple levels of a hierarchical architecture. However, the lower levels of the generative models may be habitised first, because they deal with more elemental, stereotyped and reproducible responses. With practice (e.g., elite-level sport skills), habits can include longer sequences of actions (Dezfouli and Balleine, 2012) and also possibly involve higher hierarchical levels (Pezzulo et al., 2010).

This normative view of the interactions between goal-directed and stimulus-bound actions rests on finding an optimal balance between the (computational) costs and benefits of inference. Indeed, once an action or skill is learned and is sufficiently reliable, it can be called upon with minimal processing costs and thus becomes a habit. Let us imagine that an animal is learning the contingencies between an action (say a lever press) and its consequence (say the appearance of some food that the animal can consume). When the contingency between a stimulus (the appearance of the lever), the action (pressing) and the ensuing reinforcement (consumption of the food) is reliable, the animal should stick with the habitual and reinforced lever pressing action rather than performing a model-based search for a better option. This example illustrates that a hierarchical architecture for adaptive control leads to the problem of *exploration* vs. *exploitation* (Baum, 2004, Pezzulo et al., 2013), where a model-based search (a covert form of exploration) should only be performed if its expected benefits – in terms of reinforcement – exceed its computational costs; otherwise the habitual lever pressing action should be directly exploited.

In Active Inference, free energy minimization solves this dilemma for free. Technically, the objective function in Active Inference is a free energy bound on the logarithm of Bayesian model evidence. This bound can be decomposed into accuracy and complexity. If the model is sufficient to accurately predict a succession of stimuli associated with habitual responding, then further optimization can only be achieved by minimizing complexity costs. This is exactly the same as removing (redundant) model parameters that hitherto provided top-down contextual control. Indeed, in a world where habitual responding always leads to valuable outcomes, it is suboptimal to contextualize habits – because this would lead to generalization failures and ultimately to “overfitting”, in the same way over parameterized statistical models tend to fit ‘noise’ in the data rather than capturing regularities. In other words, sensory cues are usually appropriate to trigger the right (habitual) action, without needing to postulate additional hidden or latent variables. However, occasionally cues can be misleading and thus habitual controllers would prescribe an inappropriate action. It is exactly these rare occasions that are exploited experimentally to assess whether an animal is under the control of a habitual or a goal-directed mechanism (e.g., in devaluation paradigms).

Resistance to devaluation and habitisation also illustrates how, in Active Inference, attention modulates the relative precision of the different levels of the hierarchy (Feldman and Friston, 2010). Attention can favour the integration of contextual information by increasing the precision of higher-level representations, for instance facilitating goal-directed control over a habitual response. This mechanism is important in several circumstances where contextualization becomes adaptive. Cognitive conflict occurs in behavioural paradigms such as the Stroop and the Flanker tests (Botvinick et al., 2001). In these, the goal-directed system needs to inhibit a prepotent maladaptive Pavlovian or habitual response. Cognitive conflict emerges because both low-level representations (Pavlovian or habitual) and goal-directed representations have high precision. The conflict can be resolved when attention resources increase goal-directed precision. Similarly, when an error occurs along a sequence of habitual responses, the prediction error triggers a change in expected precision and the deployment of attention to increase descending goal-directed influences, thus contextualizing habitual mechanisms and enabling corrective or remedial responses. This mechanism would explain why habitual actions (e.g., turning left at a crossroad because this is the path I take every day) could be initiated but also successively reversed by goals (e.g., after a habit-based preparation of a left turn, one can decide to turn right because today I am not going to my office). The same scheme could support *postdictive inference*; for example, an action can be initiated by habit (thus before its consequences are fully predicted) and successively a goal-based prediction can be generated to monitor and verify retrospectively that the consequences were expected and desired.

To summarize, in Active Inference, there is a unique and Bayes optimal solution for contextualizing hierarchical behaviours that is based upon optimizing expected precision – very much in the same way that we carefully evaluate the standard error of our predictions based on scientific data. The expression of these predictions, under different conditions, produces goal-directed action or habits. The implicit flexibility and context-sensitive balance is therefore based on our relative confidence in predictions at different hierarchical levels. This speaks to a possible phylogenetic trajectory of hierarchical brain architectures that subtend more sophisticated forms of control by reusing and contextualizing simpler control processes (Cisek and Kalaska, 2010, Pezzulo and Castelfranchi, 2009).

### 3.3 Learning the generative models required for hierarchical inference

In Active Inference, there is a crucial distinction between *inference* and *learning*. Inference refers to the moment-to-moment estimation of the hidden causes of sensations and corresponds to state or value estimation in standard models of behaviour. This contrasts with learning the parameters of the models predicting state transitions, which proceeds over much longer time scales and corresponds to the sort of learning considered in reinforcement and value-learning paradigms. Neurobiologically, inference can be regarded as the (Bayes) optimal updating of synaptic activity encoding expectations at different levels of the hierarchy, while learning is normally associated with the updating of synaptic efficacy or connection strengths that encode causal structure and contingencies in the world.

There are two levels of which models are acquired and optimized in Active Inference. First, the parameters of any given model can minimize prediction error averaged over suitably long periods of time; this corresponds to changes in connection strength that conform to Hebbian or associative plasticity (Friston, 2008). One interesting aspect of this (activity dependent) plasticity is that it is modulated by the precision or gain applied to postsynaptic responses (of neuronal populations encoding prediction error). In other words, prediction error is used as a teaching signal for learning – as in standard reinforcement learning models (Rescorla and Wagner, 1972, Sutton and Barto, 1998, Watkins and Dayan, 1992); however, the learning rate is mediated by precision (Mathys et al., 2011). This has the interesting consequence of assigning dopamine a modulatory role in enabling plasticity based upon prediction errors – as opposed to reporting the errors per se (see later).

The second way in which models can be optimized is to change the parameters or neuronal connections themselves – at an evolutionary or somatic (neurodevelopmental) time scale. There have also been proposals that synaptic homoeostasis (and regression) during sleep is a manifestation of model optimization (Hobson and Friston, 2012). Notice here that the model is being optimized, as opposed to the parameters or connectivity of any given model. Model optimization is a well-studied problem in statistics, where it is usually resolved through Bayesian model comparison based upon model evidence. Practically, log model evidence is usually approximated with Bayesian information criteria or variational free energy (Penny, 2012). In exactly the same way, Active Inference schemes suppose that the same (variational) free energy that is optimized by inference and learning is used to compare and select generative models in the brain. Indeed, at an evolutionary level, people have proposed free energy functionals of adaptive fitness (Sella and Hirsh, 2005). Put simply, there is a well defined (free energy) functional of sensory data that scores the quality of any generative model of environmental exchange. This functional can, in principle, be used to decide whether to add a connection or contingency to the model or, indeed, add a hierarchical level.

We have stressed that apparently distinct control systems can be gracefully integrated through hierarchical minimization of prediction error or surprise. One can generalize this theme and regard control, inference, and learning as hierarchically nested beneath model optimization at different timescales; where the imperative to minimize prediction errors (or maximize model evidence) applies universally at all levels. For example, evolution can be regarded as performing Bayesian model selection to minimize (the long-term average of) free energy, while – at a much faster timescales – our motor reflexes minimize free energy by suppressing proprioceptive prediction errors encoded by alpha motor neurons in the spinal-cord. Indeed, Active Inference has even been applied to synaptic remodelling, by considering the minimization of prediction errors at the level of the dendritic tree (Kiebel and Friston, 2011).

## 4 Summary: motivated behaviour from the Active Inference perspective

So far, we have mapped the constructs of associative learning theories onto the hierarchical generative models of Active Inference. Here, we summarize the treatment and provide a general account of *motivated behaviour*.

In the associative learning literature, motivation is often associated with the invigoration of behaviour towards salient stimuli. It acts in concert with learning mechanisms that reinforce and stabilize successful behaviours and ensures that they are emitted in the future with a higher probability (Balleine et al., 2009, Berridge, 2004). Despite a general consensus on the role of motivation in the expression and acquisition of adaptive behaviour, formal descriptions have been more elusive.

In the Active Inference view, motivated behaviour is based on the joint minimization of interoceptive and exteroceptive sensory prediction error. The first prediction error informs the current motivational need (or drive) of an organism in terms of a discrepancy between optimal homeostatic levels (e.g., hunger in terms of low glucose) and the latter specifies the sensory state or goal that the animal has to produce (by acting) to keep its homeostatic level in a viable range. Generative models of increasing complexity (Fig. 3, Fig. 4, Fig. 5, Fig. 6) provide a bridge between the motivational and procedural imperatives. The implicit sensory mappings enable the proprioceptive prediction error to be minimized as a proxy for interoceptive prediction error. In brief, deep hierarchical inference gives rise to control hierarchies that finesse and contextualize adaptive responses. For example, hunger provides a ubiquitous example of a hierarchically elaborated concept that contextualizes behaviour. In the current setting, hunger does not simply reflect an inference about hypoglycaemia but the belief that if I act in this way, I will avoid (surprising) interoceptive (low blood sugar) cues. This reflects the quintessentially counterfactual nature of allostatic processing in hierarchical models.

The thesis we pursue in this work is closely related to work on reinforcement learning controllers – and especially hierarchical controllers – for goal-directed and habitual action, and state space models (Botvinick, 2008, Botvinick and Weinstein, 2014, Daw et al., 2005, Dezfouli et al., 2014, Dezfouli and Balleine, 2012). Our primary goal was to contextualize these more recent formulations within a single (and principled) framework based upon hierarchical generative models. This (Active Inference) framework is principled because it only assumes the brain is trying to maximize the evidence for its hierarchical model of the world. All the arguments in this paper (and related formulations) follow as corollaries or necessary consequences.

Having said this, the proposed framework differs from current associative and reinforcement learning theories in several important aspects. First, in Active Inference, a unique strategy (free energy minimization) produces different modes of control under various circumstances. This stands in contrast with the view of multiple *independent* controllers (Daw et al., 2005) and suggests that the contributions of different behavioural modes are orchestrated within a coherent statistical computation.

Second, previous models postulate a qualitative distinction between different forms of control. Instead, we propose a quantitative distinction, where different controllers correspond to different levels in a continuum of abstraction – from the more abstract goal-directed representations to the simple arc reflex. Indeed, we regard the highest level of prospective reasoning – conscious and expressed in linguistic form – contextualizes a more concrete and ‘intuitive’ level of reasoning, which in turn contextualizes more elemental levels and so on, down to the lowest level of the hierarchy – the arc reflex. The level of the hierarchy that guides behaviour in a given moment (the level with the highest precision), determines *how much* goal-directed (or habitual) control is exerted over behaviour.

Thus, unlike most associative or reinforcement learning theories (Daw et al., 2005), the proposed framework does not describe habitual and goal-directed controllers as necessarily competing. Instead, they are arranged in a hierarchical scheme in which the latter contextualizes the former. There have been recent proposals that address cooperation between the two controllers (Daw et al., 2011, Keramati et al., 2011, Lee et al., 2014, Pezzulo et al., 2013) and explicit proposals that they might form a hierarchy (O’Doherty et al., 2015). Here, we are not only suggesting that the two controllers might be hierarchically arranged, but that their computations are essentially the same (acknowledging that they operate at different hierarchical levels and on different representations and contingencies). This hypothesis contrasts with the view that the human brain includes two separate systems for MB and MF control, plus an arbitration system (O’Doherty et al., 2015). In the perspective pursued here, there is only one controller that realizes both goal directed and habitual behaviour, and in which higher levels (that can be considered more goal-directed in the spectrum) contextualize lower levels (that can be considered more habitual). In this view, contextualization depends on the relative precision processing at different hierarchical levels – where, effectively, precision dynamics subsume the role of arbitration. In this setting, hierarchical arbitration emerges in a principled way from free energy minimization and conforms to Active Inference principles, in which top-down and bottom-up message passing updates predictions and prediction errors, respectively. This view stands in contrast with the proposal that hierarchical message passing involves commands, policies or reward prediction errors (Botvinick, 2008, O’Doherty et al., 2015).

By the same token, the hierarchical scheme of Active Inference differs from hierarchical reinforcement learning (Botvinick, 2008, Botvinick and Weinstein, 2014). In hierarchical reinforcement learning, an agent selects either a primitive action or a higher-level action (e.g., an option); and this hierarchical scheme can extent to many levels. In Active Inference, higher hierarchical levels influence lower levels in a top-down manner, but do not replace them. Furthermore, in Active Inference action selection and the balance between hierarchical levels depend on the *precision* of the representations at various levels, not on option- or action-specific values. These two features also distinguish the proposed framework from the hierarchical scheme of (Dezfouli et al., 2014, Dezfouli and Balleine, 2012), where the conversion from goal-directed to habitual systems depends on a “chunking” mechanism, and the reverse transformation involves “decomposing action sequences into single actions”.

Furthermore, our scheme for behavioural control is based on Bayesian inference and does not call on reward prediction errors for learning or inference. One advantage of this is the concept of reward is replaced by the realization of prior preferences. This means that *epistemic* value and *pragmatic* value (e.g. utility or reward functions) have the same currency and can be accommodated within the same (information hungry) Bayesian scheme – where this Bayesian scheme prescribes how agents can infer or learn reward contingencies (Friston et al., 2015). Also, this normative approach enables an implementation in terms of predictive coding and cybernetic processes, which have some neurobiological plausibility (see Section 5). Although there have been other proposals involving Bayesian inference and behavioural control (Friston et al., 2010, Pezzulo et al., 2013, Solway and Botvinick, 2012), none has simultaneously addressed all the controllers in the ontology of animal learning theories, as offered below.

Another distinguishing aspect is that, in this framework, actions are triggered by goals through *prediction errors*, whereas in most reinforcement learning schemes, responses are triggered by stimuli via associative links – and prediction errors have only a role in learning (e.g., using Temporal Difference learning) (Sutton and Barto, 1998). In this respect, Active Inference is closer to the cybernetic view of purposive regulation (Miller et al., 1960, Seth, 2014) than to standard associative theories.

The current formulation is also deeply connected to theories that emphasize the importance of interoceptive signals for affective processing and decision-making (Craig, 2010, Damasio and Carvalho, 2013) and especially the work of Seth and collaborators on interoceptive inference (i.e., Active Inference about interoceptive states) as a basis for emotion and conscious presence (Seth, 2013, Seth et al., 2012). Here, we take a complementary perspective and discuss the combined role of interoceptive, exteroceptive and proprioceptive inference for homeostatic control and adaptive action. By starting from different angles, all these theories highlight multilateral roles of interoception, whose implications for enactive approaches to cognition (e.g., the affective consequences of goal achievement, and the associated subjective states) remain an important challenge.

The Active Inference scheme for adaptive control connects well with hierarchical and layered robotic control architectures (Arbib, 1992, Brooks, 1991, Tani and Nolfi, 1999, Verschure et al., 2003), where higher levels encode goal-directed controllers or schemas that contextualize lower-level reflex or habit-like control loops. Some of these architectures also include a (simplified) internal physiology that grounds higher-level goals (Verschure et al., 2014). Active Inference solves the problems of action selection and arbitration between levels that are implicit in these robot architectures by using a unitary, biologically-motivated scheme: a homogeneous process of free energy minimization based on a cascade of (precision engineered) predictions and prediction errors. Within this scheme, the balance between goal-directed and habitual components of behaviour can be found in a principled way based on considerations of model model “accuracy” vs. “complexity”; see above and (FitzGerald et al., 2014, Pezzulo et al., 2013). Furthermore, Active Inference offers a principled scheme to coordinate the two ‘loops’ that, in these architectures, control physiologic needs and drives (via interoceptive signals) and goal-directed action execution (via proprioceptive and exteroceptive signals), respectively. It remains to be seen if and how these features of Active Inference architectures can be translated into more effective design principles for autonomous goal-directed robots.

This new perspective has implications for the role assigned to neuromodulators, and dopamine in particular. In reinforcement learning, phasic and tonic facets of dopaminergic discharges are assumed to encode reward prediction error (Schultz et al., 1997) and average reward expectation, respectively (Niv et al., 2007). In Active Inference, dopamine is associated with *expected precision* (Friston et al., 2012a), which sets the relative gain of ascending projections signalling prediction error (Friston, 2008),. The ensuing precision control can be understood from several perspectives. First, in a hierarchical scheme, precision regulates the predominance of each level of the hierarchy, with respect to other levels. The function of dopamine that emerges is that of balancing the relative precision of the different hierarchical levels and thus the complexity of the generative model engaged for inference. As a general rule, simulations suggest that phasic dopamine firing elevates the precision of beliefs (probability distributions) over competing and hierarchically composed policies; thereby enabling more precise action selection. This can explain the empirical evidence that higher postsynaptic dopamine availability is associated with enhanced motor vigour, for instance speeding up reaction times (Berridge, 2004, Salamone and Correa, 2012), and with a very fast ‘habitisation’ of behaviour (Gremel and Costa, 2013). Also, this can explain the observation from several cognitive tasks of an inverted-U function, relating dopamine levels to performance (Cools and Esposito, 2011). This would be the consequence of an optimal dopaminergic activity, which would correspond to the engagement of the level of the generative model with the best speed/accuracy trade-off. The final point speaks to an important issue; namely that the precision or confidence about future behaviour can itself be optimized with respect to free energy or model evidence. In other words, there is an optional precision of beliefs about any choice behaviour under uncertainty.

**Fig. 7:**
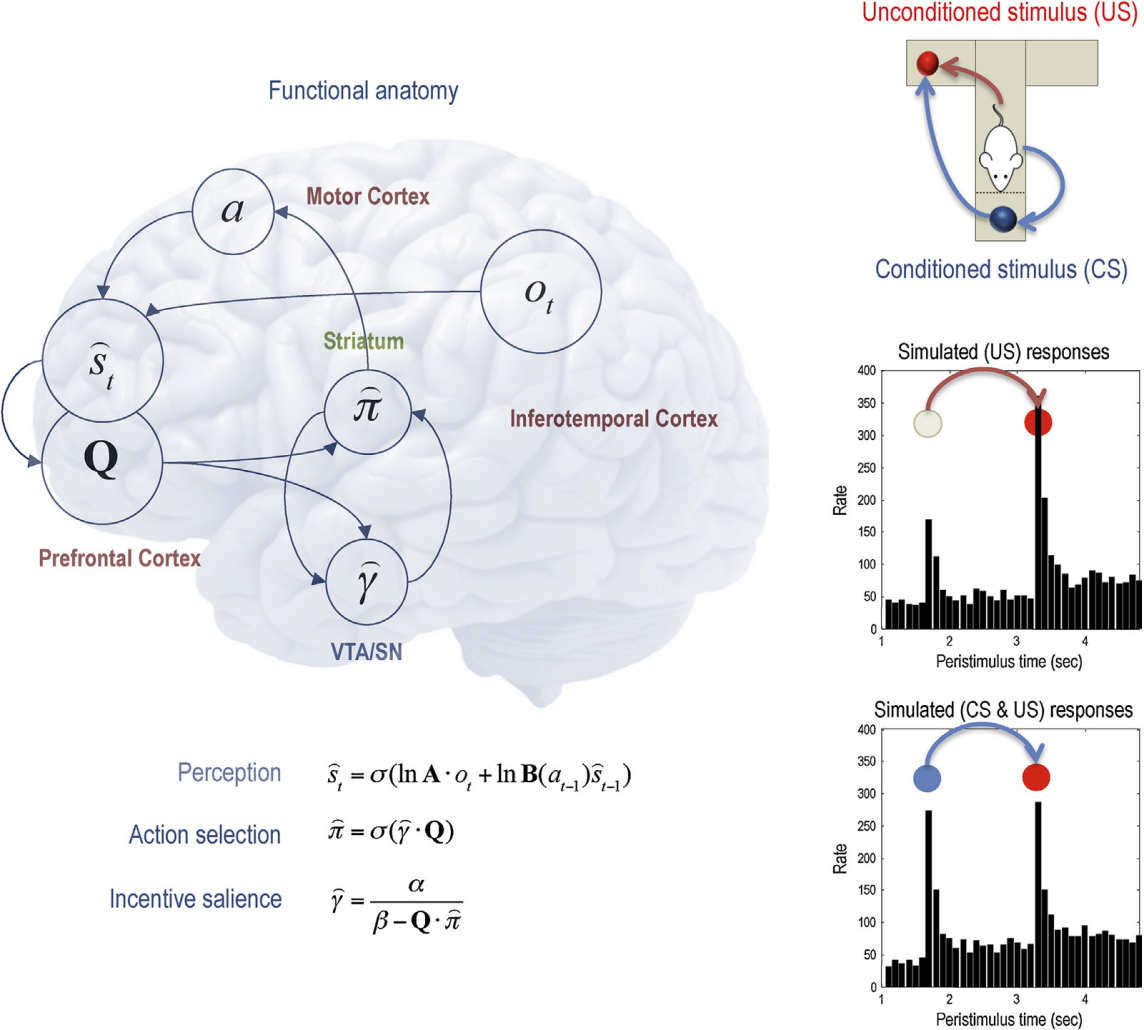
This figure illustrates the functional anatomy implied by a simple message passing scheme based on variational Bayes, and generative models based upon Markov decision processes; see (Friston et al., 2014) for details. It includes the following variables: observations (*o_t_*), expected states of the world (${\overset{⌢}{s}}_{t}$), action (*a_t_*), expected action sequences or policies ($\overset{⌢}{\pi }$) and their precision ($\overset{⌢}{\gamma }$). *Q* represents the quality of a policy scored in terms of its (epistemic) value or expected free energy. The equations corresponds to (variational) Bayesian updates, where *A* and *B* are probability transition matrices mapping hidden states to observations and hidden states to hidden states under different actions respectively. *σ* is a softmax function. Left panel: here, we have associated the Bayesian updates of hidden states of the world with perception, control states (policies) with action selection and expected precision with incentives salience. Right panel: this shows the results of a simulation in terms of simulated dopamine discharges, of the kind that is usually associated with reward prediction errors (Schultz et al., 1997), but which can also be modelled under an Active Inference scheme. The key thing to note is that the responses to an informative cue (or conditioned stimulus CS – blue) pre-empt subsequent responses to the reward (or unconditioned stimulus US – red). In this simulation, the agent was shown a cue that resolved uncertainty (i.e., had epistemic value) about where to find a reward in a simple T-maze (upper right panel). In this context, dopaminergic responses appear to transfer from the US when it is encountered without (middle right panel) and with (lower right panel) a preceding CS.

In Active Inference models of choice behaviour (Friston et al., 2014, Friston et al., 2013), precision reports opportunities to achieve a goal (or more precisely, confidence in the opportunities) that invigorate and select action; see Fig. 7. The expected precision or confidence about preferred outcomes reports on the progress towards the goal – like spatial proximity or sub-goals (Lepora and Pezzulo, 2015, Maisto et al., 2015) – and in accordance with early cybernetic models of purposive action such as the *Test-Operate-Test-Exit (TOTE)* model (Miller et al., 1960) precision can be updated (in a Bayes optimal fashion) during goal-directed action – rather than just mediating learning by reporting reward prediction errors. This view is compatible with theories that describe dopamine function within a “wanting” system (Berridge, 2004) that is not hedonic (as in the “liking” system) but instrumental to goal pursuit (Salamone and Correa, 2012).

Finally, precision regulates the gain of (postsynaptic) prediction errors that drive associative (synaptic) plasticity (Friston, 2008). The post-synaptic effects of dopaminergic projections from the ventral tegmental area and substantia nigra varies across target regions (e.g., different portions of basal ganglia and cortex), due, for instance to the complex role of receptors such as D1 and D2 (Clark and White, 1987). This prompts the hypothesis that the effects of increased/decreased dopamine activity vary within and across individuals – an idea that has inspired interpretations of several symptoms and signs in neuropsychiatric disorders. For example, there is much current interest in understanding a failure to attenuate sensory precision as an explanation for several symptoms and signs in neuropsychiatric disorders (Edwards et al., 2012, Friston et al., 2012a).

Fig. 7 illustrates how a hierarchical scheme can explain one of the cardinal features of dopaminergic discharges. In this example, we used a T-maze to present conditioned stimuli (CS) in the form of cues that indicated which arm was baited with a reward. Using a simple Bayesian (variational) belief update scheme (see figure legend) it is fairly simple to reproduce dopaminergic discharge profiles that are similar to those observed empirically. In brief, the confidence about subsequent behaviour is encoded by dopamine (whose phasic discharges increase the precision of probabilistic beliefs about competing policies). The figure compares (simulated) dopamine responses to the CS and unconditioned stimulus (US or reward) before the agent knows where the reward is and after it has inferred its location. This example shows that hierarchical inference offers a sufficient explanation for dopamine responses in terms of the confidence or precision about subsequent behaviour. Here, the CS has epistemic value because it resolves uncertainty about subsequent (goal directed) behaviour. Under these circumstances the CS elicits a burst of dopamine firing that is effectively transferred from the US to the CS. See Friston et al. (2015) for details of this particular example.

A final important point concerns the relationship between drives and goals. In most psychological theories, drives are usually linked to basic motivations and homoeostasis (e.g., interoceptive hunger or thirst), while goals are considered to have more elaborate (exteroceptive) sensory and cognitive aspects (e.g., dining in a restaurant), but their relations are often unclear from a mechanistic viewpoint (Pezzulo et al., 2014b, Verschure et al., 2014). In Active Inference, goals are ultimately grounded in expectations about physiological allostasis, and engage a cascade of interoceptive, proprioceptive and exteroceptive loops. The objective is to suppress *interoceptive* prediction errors (Barrett and Simmons, 2015, Pezzulo, 2013, Seth et al., 2012) through action, which in turn requires bodily signals to be represented centrally and controlled through a deep hierarchical modelling that – crucially – engages *proprioceptive* and *exteroceptive* loops. This implies that at every level of the hierarchy, goals of different complexity are represented that can be distally (and evolutionarily) related to homoeostasis but at the same time are (conditionally) *independent* from them. Here, conditionally independent means that goals have autonomy in steering and controlling behaviour; for example, the goal of ‘going to a fancy restaurant’ is certainly linked to the drive to eat (or socialize) but it can induce restaurant-searching behaviour in the absence of hunger. Furthermore, going to a restaurant does not in itself cause a reversal of hypoglycaemia. The ‘autonomisation’ of goals from primary drives is a key feature of higher animals like humans (Pezzulo and Castelfranchi, 2009) and becomes more evident in pathological conditions, where certain goals such as gambling can become pathological and maladaptive (Montague et al., 2012). Autonomy is a general characteristic of hierarchically organized architectures that, necessarily, embody conditional independencies and enable organized behaviour over extended timeframes.

This account thus goes beyond purely Hullian, drive-minimization theories of motivation and emphasizes that an important part of motivated behaviour is directly *guided by goals* and internal representations of desired future states. Basic drives associated with evolutionary imperatives (e.g., good levels of glucose) naturally constrain the acquisition of new empirical priors that define goals at some (high) level of an agent's generative model. In turn, these high-level priors contextualize behaviour that is internally consistent with lower-level drives provided they are afforded sufficient precision. Although the ultimate reason for the emergence of goals is that they satisfy some internal drive, the activation of a specific goal might or might not be caused by concurrent interoceptive inference. Thus, the goal system supports and – in a certain sense – supersedes the drive system: (Montague, 2006) speculates that the goal system capitalizes on existing brain architectures for (reward) prediction errors and reuses it in an open-ended way. In a similar vein, Passingham and Wise (2012) discuss the adaptive advantages of the more advanced (prefrontal) goal systems. They argue that the (new) prefrontal areas of anthropoid primates extended the (older) reinforcement-learning system, increasing its flexibility and adaptability; for example, by generating (foraging) goals from single learning episodes. The resulting architecture for motivated behaviour frees higher animals from the immediate demands of homeostatic regulation. While this may predispose to maladaptive behaviour, it also permits an open-ended proliferation of goals and desires that characterize our human lives – because, as Baruch de Espinoza puts it, “desire is the essence of a man”.

## 5 Simplified functional anatomy of hierarchical Active Inference

**Fig. 8:**
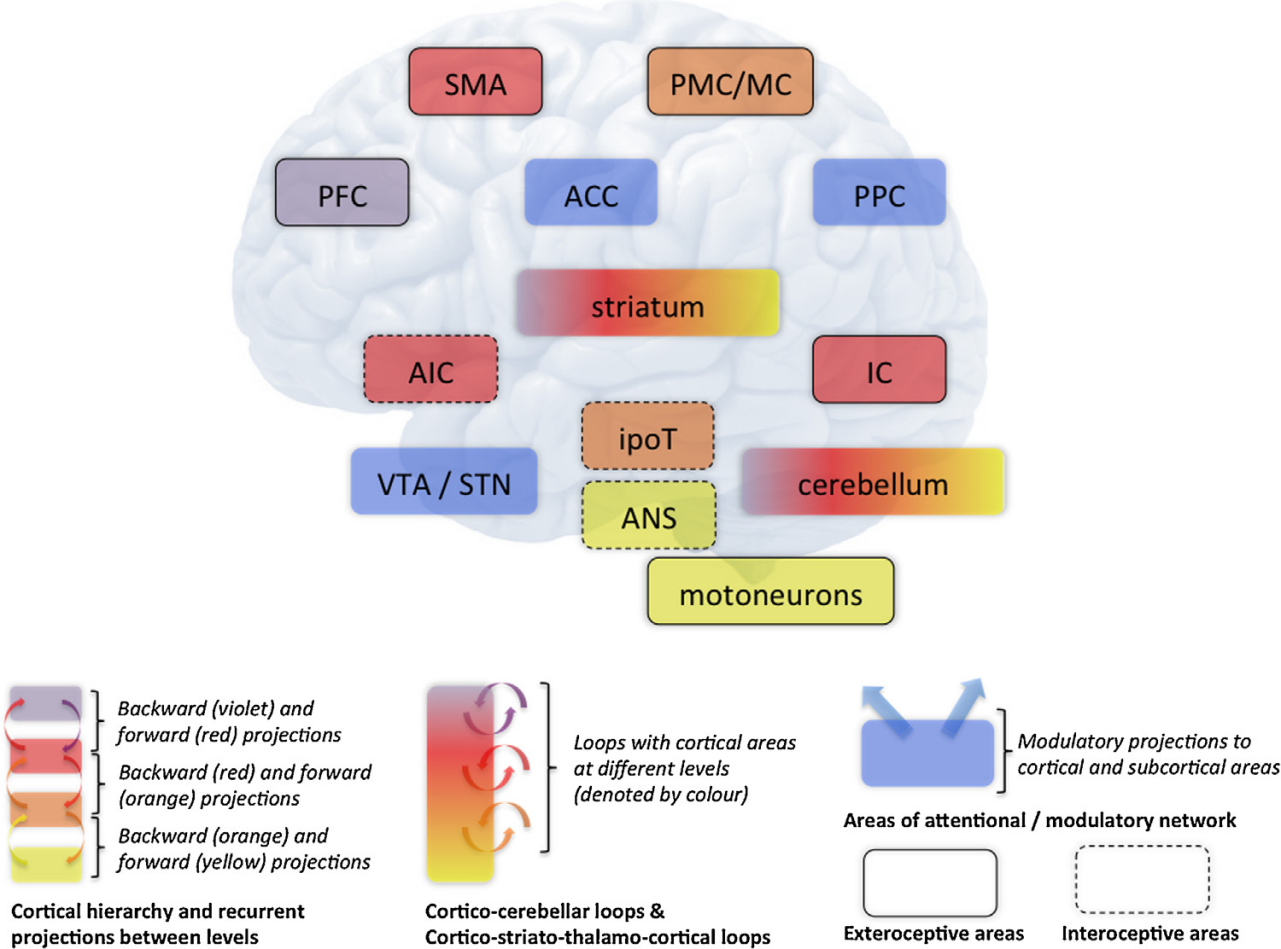
Simplified functional anatomy of hierarchical Active Inference. Upper panel: different levels of the hierarchy are represented by a colour gradient from violet (the highest level: prefrontal cortex, PFC), red (supplemental motor area, SMA; Inferotemporal cortex, IC; anterior insular cortex, AIC), orange (premotor/motor cortex, PMC/MC; striatum; ipothalamus, ipoT) to yellow (autonomous nervous system, ANS; motoneurons). Blue areas (anterior cingulate cortex, ACC; posterior parietal cortex, PPC; and the dopaminergic ventral tegmental area and substantia nigra, VTA/SN) are portrayed as part of an attentional network that modulates the relative precision of different levels in the hierarchy. Lower panel: simplified schematic of the connections between brain areas of the upper panel. The lower-left panel shows the recurrent projections between brain areas at different hierarchical levels (denoted by colour), and follow a predictive coding scheme: backward connections (e.g., from PFC to SMA) convey descending predictions and forward connections (e.g., from SMA to PFC) pass prediction errors. The lower-middle panel shows the loops between cortical and subcortical brain areas at different hierarchical levels, which include loops between nucleus accumbens/ventral striatum and orbital/ventral PFC; between caudate/dorsomedial striatum and prefrontal/parietal association cortices; and between putamen/dorsolateral striatum and sensorimotor cortices (Yin and Knowlton, 2006). The lower-right panel exemplifies the modulatory projections the attentional network to various cortical and subcortical brain areas (e.g., from VTA/SNT to PFC and striatum); and the distinction between areas that form exteroceptive and interoceptive networks.

A simplified functional anatomy for the hierarchical Active Inference scheme that we have discussed is shown in Fig. 8. This should be considered as a schematic that organizes ideas about the functional anatomy underlying active or embodied inference. This sort of scheme should not be considered as a comprehensive characterization of functional brain architectures: many components are depicted very simplistically – and some brain areas are not discussed at all. However, it may be useful as a general framework about the functional neural organization and as a working hypothesis for the interpretation and testing of empirical studies. Here anatomical loops of increasing complexity are proposed to embody the ‘gradient’ of control discussed earlier, with the more elaborated goal-directed control loops that contextualize the more elemental sensorimotor constructs within generative models of increasing hierarchical depth. The different levels of the Active Inference hierarchy are represented by a colour gradient from violet (the highest level: prefrontal cortex, PFC), red (supplemental motor area, SMA; Inferotemporal cortex, IC; anterior insular cortex, AIC), orange (premotor/motor cortex, PMC/MC; striatum; ipothalamus, ipoT) to yellow (autonomous nervous system, ANS; motoneurons). Connections among areas represent recurrent projections between areas that follow the usual logic of *predictive coding*, where backward connections convey descending predictions and forward connections pass prediction errors.

Hierarchically higher areas play a more prominent role in the goal-directed system, whereas orange areas predominate in the Pavlovian and habitual systems. The central position assigned to prefrontal areas at the apex of the goal-directed system is in accordance with a large literature highlighting its importance for flexible cognitive control and executive function (Fuster, 1997, Miller and Cohen, 2001). Evidence is consistent with the hypothesis that contextual representations of increasing abstraction and timescale are processed along a posterior-anterior axis in PFC, suggesting that this region itself is hierarchically organized (Koechlin and Summerfield, 2007). Goal-directed control requires the *integration* of information from different sources. This corresponds in our model to the fact that the exteroceptive network (including SMA, IC, striatum, MC and motoneurons – bold circles) is anatomically segregated from the interoceptive network (including AIC, ipoT and the ANS – dotted circles), but the two are integrated at the multimodal highest level in the PFC, see Fig. 2 and Gu et al. (2013). Goal-directed control also requires knowledge of the contingencies between actions and outcomes (Balleine and Dickinson, 1998). This form of knowledge is encoded in various ways – and at increasingly longer timescales – in motor, premotor, and prefrontal brain areas, which overall form hierarchical control loops.

Another brain structure with a primary role in goal-directed control is IC and the closely related hippocampus. The ability of the hippocampus to support goal-directed learning and behaviour has been studied principally in spatial navigation (Buzsáki and Moser, 2013), but evidence suggests that this area also supports other advanced cognitive abilities that require prospection, such as imagination of fictive scenarios (Hassabis and Maguire, 2009). From the associative learning perspective, the hippocampus has been linked to a form of instrumental process called ‘episodic’ control. Specifically, the hippocampus is thought to store state-action sequences that successfully attain a goal and then replay those sequences when a constituent state is encountered (Lengyel and Dayan, 2008). This ability to generate fictive sequences of states (Johnson and Redish, 2007, Pfeiffer and Foster, 2013) has been interpreted in terms of reactivation in the context of generative models (Penny et al., 2013, Pezzulo et al., 2014a, Pezzulo et al., 2013). Furthermore, the hippocampus has been consistently linked to memory consolidation (Buzsáki, 1996), a role that can be characterized within Active Inference as the reporting of the expected information, precision and novelty of sequential events (Strange et al., 2005). In short, the hippocampus is an important nexus for both the expression of goal-directed behaviour (possibly via the covert replay of previous experiences encoded in the generative models) and its acquisition (possibly by signalling the opportunity to optimize or revise generative models).

In the hierarchical architecture depicted here, brain structures controlling more complex strategies – that are mandated by goal-directed behaviour and cognitive control – do not replace more ancient brain structures underlying the simpler strategies based on fixed stimulus-response pairs, but contextualize them. In this way, more elaborate forms of action control orchestrate more elementary sensorimotor architectures that support situated action or *affordance competition* (Cisek and Kalaska, 2010). In the present scheme, this may involve cortical (motor and premotor) and subcortical regions, such as the striatum and hypothalamus.

A widespread view is that different striatal territories (coupled with limbic, associative and sensorimotor cortex) participate in distinct cortico-subcortical loops – limbic, associative, and sensorimotor, respectively – and support different aspects of behavioural control (Yin and Knowlton, 2006). For example, the dorsolateral striatum is thought to be more involved in stimulus-response learning and habitual control, the dorsomedial striatum has been linked to action-outcome learning and goal-directed action, and ventral striatum to Pavlovian values (Glascher et al., 2010, Liljeholm and O’Doherty, 2012, Mannella et al., 2013, O’Doherty et al., 2004). In a related perspective, these striatal territories might compute outcome predictions in parallel using different kinds of information as input (Pennartz et al., 2011). In this perspective, the model-based vs. model-free dichotomy is replaced by a more nuanced view, in which striatal territories embody different predictors (or models): from the more complex outcome predictions in ventral and dorsomedial striatum (analogous to model-based mechanisms), to the simpler predictions about actions based on somatosensory and motor information in the dorsolateral striatum (analogous to model-free mechanisms). The idea that different striatal territories support different aspects of behavioural control is coherent with the Active Inference scheme where generative models are not purely cortical structures but involve cortico-subcortical loops. In the perspective pursued here, however, the striatal subdivision is conceived as in hierarchical terms, rather than in terms of competition among parallel controllers or predictors. This hierarchical view of striatal computation is indeed supported by recent neurophysiological evidence (Ito and Doya, 2015).

Brain loops might extend beyond the striatum and include, for example, the cerebellum, which – given its remarkably homogenous microarchitecture – has been often assumed to be able to encode a large repertoire of internal (generative) models required to predict and control action and its timing (Caligiore et al., 2013, Imamizu et al., 2003, Miall and Wolpert, 1996, Wolpert et al., 1998). Embodied views of cognition suggest that the re-enactment of the same perceptual-motor loops – and internally generated brain dynamics – might realize increasingly complex cognitive functions such as planning, imagery, and conscious thought (Buzsáki et al., 2014, Grush, 2004, Hesslow, 2002, Jeannerod, 2001, Pezzulo, 2011, Pezzulo and Castelfranchi, 2009).

Motoneurons and autonomic effectors are found at the lowest level of the hierarchy. These infrastructures constitute the basic building blocks to enact arc reflexes and are exploited and contextualized by the higher hierarchical levels. It is worth noting that in the Active Inference scheme, motor areas represent proprioceptive predictions and not motor commands (as traditionally assumed), in the same way as sensory (e.g., visual) areas represent exteroceptive predictions (Adams et al., 2013). Motoneurons are responsible for activating arc reflexes that fulfil these predictions, thus guiding overt action. They are thus the locus where predictions are unpacked in a kinematic frame of reference and transformed into overt action.

In addition to the higher-to-lower control hierarchy, the Active Inference scheme requires an attentional network that modulates the relative precision of different levels in the hierarchy. In Fig. 8 this role has been assigned to the blue areas (anterior cingulate cortex, ACC; posterior parietal cortex, PPC; and the dopaminergic ventral tegmental area and substantia nigra, VTA/SN). We hypothesize that ACC and PPC (linked preferentially with higher order areas through recurrent connections) are involved in modulating the relative precision of PFC relative to lower level areas; whereas VTA/SN (projecting widely to all other areas) is more important in regulating the precision of exteroceptive and interoceptive sensations (blue connections). The attentional network has an important role in the balance between more cognitively complex goals and more elementary ones, maintained at different hierarchical levels, which is an important hallmark of cognitive control (Fuster, 1997, Miller and Cohen, 2001). Here, cognitive control entails a high degree of precision of “higher” areas such as PFC, so that more cognitively complex and long-term goals dominate the inference (Pezzulo, 2012, Stoianov et al., 2015). However, in some cases, such as in drug addiction, some aspects of prefrontal function decline and the agent becomes unable to contextualize or suppress (hierarchically lower) cortico – dorsal striatum loops that implement habits (Belin et al., 2009).

In sum, we propose a hierarchical neural architecture that extends from cortical areas to peripheral reflexes. This hierarchy is organized according to the level of abstraction of representations embodied by the different areas. The neural architecture is based on *predictive coding*, which constitutes a Bayesian inference machine that guides both perception and action.

### 5.1 Empirical evidence supporting the proposed framework and novel predictions

Active Inference rests upon, and extends, a predictive coding scheme, which provides an explanation for many aspects of functional brain architectures. In brief, it explains the hierarchical nature of cortical connections; the prevalence of backward connections and explains many of the functional and structural asymmetries in the extrinsic (between region) connections that link hierarchical levels (Zeki and Shipp, 1988). These asymmetries include the laminar specificity of forward and backward connections, the prevalence of nonlinear or modulatory backward connections (that embody interactions and nonlinearities inherent in the generation of sensory signals) and their spectral characteristics (Adams et al., 2013, Bastos et al., 2012, Friston, 2008). The specific evidence for predictive coding in the motor system is reviewed in (Shipp et al., 2013).

The studies mentioned above all provide some indirect support to the framework for adaptive behavioural control proposed here, which implements the plausible predictive coding and Active Inference schemes. Furthermore, this new framework allows to explain previous evidence and to make novel predictions that rest on our central premise; namely, that habitual and goal-directed behaviours are context-sensitive expressions of the same hierarchical (active) inference. In other words, by definition, habitual behaviours become habitised by virtue of selecting low-level hierarchical contingencies to make proprioceptive and interoceptive predictions that induce behaviour. The selection rests upon modulatory (gain) control that we presume is reflected in dopaminergic activity. Therefore, neuromodulatory (e.g., dopaminergic) systems should be responsible for hierarchical selection – or mixture – of goal directed or habitual behaviour.

Empirical studies suggest that dopaminergic projections are divergent and modulate, in a complementary fashion, higher and lower hierarchical levels of executive (corticostriatal-thalamic) processing. Our proposal that dopamine encodes the relative precision of the different hierarchical levels fits with such evidence. This divergence and modulatory aspect has been discussed under RL models (O’Doherty et al., 2015). However, we stress that, in the current view, dopamine does not encode reward prediction errors but the precision of prediction errors at various levels of a hierarchy. Because precision is a ubiquitous attribute of all (hierarchically deployed) prediction errors, its computation and broadcasting mandates a convergent-divergent neuroanatomy and a modulatory neurophysiology. Because, in Active Inference, optimization rests upon reciprocal message passing, dopaminergic reference to multiple hierarchical levels must be reciprocated by (monosynaptic or polysynaptic) afferents from these regions.

Although other schemes for hierarchical control have been proposed that use belief precisions or related constructs to modulate the balance of model based and model free controllers (Daw et al., 2005, Gershman and Daw, 2012, Lee et al., 2014, Pezzulo et al., 2013), there are some unique features of the Active Inference scheme that make novel and empirically testable hypotheses. For example, in Active Inference, but not in the aforementioned models, the optimal value of the precision parameter (in terms of free energy minimization) is computed on-line as part of inference. Furthermore, this precision controls the degree of “exploration” of the system, because, formally, it corresponds to the temperature parameter of softmax choice rules. In other words, the degree of exploration is derived from first principles rather than being tuned to behaviour as an ad hoc parameter. This feature of the model entails the novel prediction that a higher precision or confidence in their behavioural plans makes animals less exploratory and thus ultimately produces more stereotyped responses, which again speaks to habitisation. By the same token, exploration is favoured by the presence in the environment of cues that have *epistemic value* and can improve the precision of the behavioural policies (Friston et al., 2015).

Furthermore, an idea specific to our framework is that habitual and goal-directed behaviour form a continuum rather than a strict dichotomy, and can cooperate rather than just compete. Consistent with this view, recent behavioural and neural evidence supports the idea that at any given moment action selection results from a mixture of goal-directed and habitual control, rather than being under the exclusive control of either of the two systems (Daw et al., 2011, Lee et al., 2014, Otto et al., 2013). Although these data can still be interpreted as resulting from two systems working in parallel and competing at a later stage, a much simpler explanation is the idea of a unitary hierarchy scheme in which the different levels correspond to specific degrees of complexity. In this perspective, here the novelty of the proposal lies in the architectural scheme itself, which does not foresee multiple or even modularized controllers that perform heterogeneous computations plus an arbitration mechanism, but a unique architecture that performs homogeneous computations at all hierarchical levels.

Strictly speaking, in the proposed scheme, habitual behaviour is not completely model free in that it continues to depend on the (simplest) type of predictive model, of the kind “because there is a stimulus, I expect a response”. In Active Inference, it is the expected response that enslaves action, thus even these simple responses are prediction-based. Neurophysiologically, this is compatible with the view that the dorsolateral striatum, which has been long associated with habitual control, encodes simple models that generate predictions based on the animal's somatosensory and motor information (Pennartz et al., 2011). This scheme lends itself to a straightforward hierarchical extension, in which simpler models can be successively contextualized and can incorporate elements of goal-directedness, but only when more complex models at the higher hierarchical levels have sufficient precision to exert an influence. In other words, in this scheme there is no need to see habitual and goal-directed behaviours as regulated by distinct or modular processes.

The key ethological insight afforded by hierarchical Active Inference is that all behaviours are hierarchically contextualized. Not only does this predict that goal-directed and habitual responses can be mixed, but also the specific conditions where this should occur. For example, it predicts that responses should fail to be contextualized – and thus become more habitual – when the system is ‘taxed’ by concurrent cognitively demanding tasks, a phenomenon that has been often interpreted in terms of reinforcement learning theories (Gershman et al., 2014, Otto et al., 2013). Furthermore, it predicts that under certain conditions habitual behaviour can cease to be purely ‘model free’, thereby disclosing evidence of its context sensitivity. For example, phenomena like extinction and the context-dependent reactivation of extinguished associations can be explained in terms of the (hierarchical) selection of learned associations at different periods of exposure to the environment. For example, the extinction of a conditioned response (say, a conditioned response of hunger and salivation following the sound of a bell) would not derive from an attenuation of the sound- food association, but from the fact that the response can be contextualized by a (higher) mechanism that learns how the probability of food availability depends on the sound, in different contexts. In the same way, the response can be reactivated when the right contextual conditions are detected, thus providing the top-down mechanisms enough precision to influence or overcome any behavioural habit developed in the interim; see also (Gallistel, 2012).

Another set of predictions stem from the idea that action selection and initiation depends on (multimodal) prediction errors, not only stimuli. This view is compatible with a large body of evidence in the ideomotor theory, according to which actions, including those that are as simple as button presses (as used in most human studies), are planned and selected based on their anticipated effects (Hoffmann, 2003, Hommel et al., 2001, Kunde, 2003, Kunde, 2001, Prinz, 1997; Schuetz-Bosbach and Prinz, 2007). By expanding this framework to consider a role for interoceptive (not only exteroceptive or proprioceptive) prediction errors, it may be possible to address the link between interoceptive states and action selection and initiation, which is currently under-explored in the animal learning literature. Two caveats for experimental studies investigating this link would be the largely anticipatory nature of interoceptive events and the fact the relations between actions and interoceptive consequences can be distal and mediated by other (exteroceptive or proprioceptive) events, see Section 3.1.

To summarize, in this section we have presented a range of empirical predictions that stem from our framework. Some of these predictions are unique to this framework and could serve to disambiguate it from alternatives. Other predictions, have been addressed under other normative perspectives; most notably, reinforcement learning. Perhaps the most important (novel) contribution of our proposal is that it accommodates multifarious phenomena, which have been addressed using different – and often incompatible – computational approaches. Furthermore, the framework can call upon specific process theories that are biologically plausible; for example, predictive coding and cybernetic schemes (Friston, 2010). In other words, there is the potential for an explicit connection between the normative (approximate Bayesian inference) level and the processes underlying neurobiological implementation.

## 6 Conclusions

In this article, we have cast “homoeostasis” and “behavioural control” in terms of Active Inference. Under this perspective, drive and goal achievement mandate the suppression of prediction errors of different kinds (interoceptive, proprioceptive, and exteroceptive) within a hierarchical architecture, and their resolution through action. The underlying computations are driven by a unique imperative: free energy minimization. However, when the same free energy minimization is expressed in different conditions (e.g., before, during or after learning of generative models) they produce different forms of behavioural control that correspond to reflexes, conditional responses and goal-directed behaviour.

This paper establishes a link between Active Inference and associative learning theories of animal behaviour, with potential benefits for both. On the one hand, the link extends the scope of Active Inference to the self-regulation of bodily (as opposed to sensorimotor) states and interoceptive inference (Pezzulo, 2013, Seth et al., 2012). Furthermore, the link with animal learning theories clarifies the nature of hierarchical inference. For example, empirical priors in generative models correspond to “drives” and “goals” in the animal learning literature – despite the fact they are called “beliefs” in the Bayesian brain literature. We have suggested that an initial set of priors might correspond to Pavlovian values shaped by evolution, while others are acquired by learning generative models. On the other hand, the connection with Active Inference suggests that the behavioural controllers proposed in the animal learning literature might be seen as the successive contextualization of simpler sensorimotor mechanisms in hierarchical generative models. This view emphasises an inclusive and unified view of functional brain architectures, rather than a collection of distinct and separable modules.

The homeostatic view proposed here clarifies why Active Inference agents are not plagued by the (infamous) “dark room” problem (Friston et al., 2012b). Homeostatic regulation implies a continuous update of empirical priors for action, whose dynamics are dictated by the uninterrupted flux of interoceptive message passing between brain and body. This flux incessantly supplies new set points or goals – and a “moving target” for behaviour. Thus, any organism equipped with a body must face reality rather than live in a darkened room. It is exactly in this sense that (Pavlov, 2010) argues that the brain provides the interface between the internal milieu of the body and the external milieu of the environment. In other words, the interoceptive state is what organisms try to maintain within a viable range – because departures can be dangerous. Exteroceptive states are conserved, provided this sufficient to keep interoceptive states in a physiological range – but here more variability and “exploration” is allowed. Maintaining this delicate balance is an important constraining factor for any robust control system, biological or artificial.

Our analysis also grounds the abstract computations of *free energy minimization* as described in theoretical neuroscience (Friston, 2010). It suggests that free energy can be minimized at multiple timescales: evolutionary for Pavlovian values, lifelong learning for instrumental control (in particular habitual control), and on-line search-based computations for goal-directed control. In this perspective, the same free energy computations that optimize on-line action might also be used to describe how brain structure is optimized during evolution to embody an organism's needs and how it is adapted during development to retain useful (habitual) strategies and to increase the precision of lower hierarchical layers. These elements are all implicated in free energy principle but here we provide a new perspective on the mechanisms supporting that minimization and the timescales at which they operate.

Our review of different approaches to behavioural control highlights that some terms are used in different ways; for example, the concept of “value”. In the homeostatic view pursued here, value is the complement of surprise or prediction error (because maintaining homeostasis is “valuable”). This implies that any state or action can have an associated value if it links, directly or indirectly, to the minimization of prediction error. Essentially, all adaptive systems should pursue value through action in some way.

It is important to note that although value computations are implemented differently in different systems, they essentially use the same logic, which defines the normative aspect of these behaviours. In most homeostatic theories, the value is pursued directly by engaging (Pavlovian) innate approach or avoidance actions. In reinforcement learning, for instance a temporal-difference reward prediction error is used to train (habitual) controllers that can be considered a proxy for achieving a certain “reward”, which is in turn a proxy for restoring good homeostatic values. In episodic controllers, action sequences that lead to reinforcement are stored and re-enacted in similar circumstances. In Active Inference, free energy minimization can either consider sensory prediction errors directly, or do so indirectly by considering proxies (e.g., prediction errors at higher levels of hierarchical models that link sensory prediction errors over modalities and time). In short, all these approaches rest on similar or even identical value computations, but differ in specific aspects of their mechanistic interpretation and implementation.

Understanding the computational and neuronal basis of motivated behaviour is a key objective of many disciplines including psychology, neuroscience, and (neuro)economics. The framework offered here provides principled and mechanistic hypotheses about how the basic brain design of simpler animals might have been reused and extended through generative models and, while retaining essential *embodied* aspects, permits higher animals to achieve open-ended goals.

## Figures and Tables

Figure adapted from Gu et al. (2013).
